# The Types of Psychosocial Factors Associated with Suicidality Outcomes for People Living with Bipolar Disorder: A Scoping Review

**DOI:** 10.3390/ijerph21050525

**Published:** 2024-04-24

**Authors:** Robert C. Dempsey, Alyson L. Dodd, Patricia A. Gooding, Steven H. Jones

**Affiliations:** 1 Department of Psychology, Faculty of Health and Education, Manchester Metropolitan University, Manchester M15 6BH, UK; 2 Department of Psychology, Faculty of Health & Life Sciences, Northumbria University, Newcastle upon Tyne NE1 8ST, UK; 3 Division of Psychology & Mental Health, School of Health Sciences, Faculty of Biology, Medicine and Health, University of Manchester, Manchester M13 9PL, UK; 4 Spectrum Centre for Mental Health Research, Faculty of Health and Medicine, Lancaster University, Lancaster LA1 4YW, UK

**Keywords:** bipolar disorder, suicide, suicidal ideation, suicide prevention, psychosocial, etiology, trauma, impulsivity

## Abstract

Bipolar Disorder is associated with high rates of suicidal thoughts, behaviors, and outcomes, yet the lived experience of suicidality and Bipolar Disorder is not particularly well understood. Understanding the role of psychosocial aetiologies in suicidality outcomes for those living with Bipolar Disorder is key for developing appropriately targeted interventions focusing on factors that are amenable to change. In line with PRISMA guidance, we conducted a scoping review to identify the types of psychosocial factors studied in relation to the experience of suicidality for people living with Bipolar Disorder diagnoses. Systematic literature searches identified a sample of 166 articles from which key study data were extracted and charted. A narrative synthesis of the reviewed literature is presented ordered by the factors investigated across studies, a frequency count of the types of psychological/social aetiologies studied, and a brief overview of the key findings for each aetiology. Most of the identified literature took the form of quantitative cross-sectional studies, with only one qualitative study and 18 quantitative prospective studies. The most studied aetiologies were trauma (specifically early adverse experiences and childhood traumas) and stressful life events, impulsivity (primarily subjective self-reported trait impulsivity), social support and functioning, and personality/temperament factors. Only six studies in the final sample reported basing their research questions and/or hypotheses on an explicit theoretical model of suicide. The literature was primarily focused on using self-report measurements of key aetiologies and on factors which lead to worsened suicidality rather than focusing on potentially protective or buffering factors. Future research needs to better justify the aetiologies investigated in relation to suicidality outcomes for people living with Bipolar Disorder, including a firmer basis in theory and hypothesis testing, more prospective designs, and the use of alternative assessments of psychosocial aetiologies in addition to self-report questionnaires.

## 1. Introduction

Bipolar Disorder is associated with some of the worse outcomes and rates of suicide across all clinically recognized mental health conditions [1,2]. Rates of death by suicide amongst people with a Bipolar Disorder diagnosis are estimated to be 20–30 times greater than those found in the general population [3,4]. Other estimates suggest that around 19% of people living with Bipolar Disorder die by suicide and between 20–60% attempt suicide [5,6]. Understanding the factors implicated in the experience of suicidal thoughts and acts in people with Bipolar Disorder is therefore crucial for identifying those at risk and in developing appropriately targeted interventions.

Numerous risk factors for suicide for people living with Bipolar Disorder have been identified. Suicide risk, however, is complex and can be influenced by a combination of biological, social, environmental, psychological, and clinical factors [7]. Common risk factors for suicide for people with Bipolar Disorder include the severity of depressive symptoms, past suicide attempts, substance abuse, mixed mood episodes, mood episodes at first onset, family histories of Bipolar Disorder, divorce, and relationship breakdowns [8,9].

Whilst such risk factors may identify broader groups at an elevated risk for suicide, such approaches tend to identify factors which have low specificity in predicting who may die by suicide [10]. These risk factors also do not adequately distinguish between the risk for suicidal thinking and behaviors [11] or detail the processes or pathways to suicide [12]. Risk factor-based approaches may also not accommodate more individualized approaches to understanding peoples’ experiences and their actual suicide risk [1]. Such risk factors may also combine with more psychosocial mechanisms and factors to determine an individual’s suicide risk. There is also a growing recognition that distinct factors may be implicated in different points of the suicidal ideation-to-enaction process, and that suicidal thoughts and acts experienced by people with Bipolar Disorder may be the result of a complex series of processes involving many biopsychosocial factors [2].

The dominant focus on identifying common risk factors for suicidal behaviors and attempts means that there is an emphasis on current mood and related psychiatric symptom assessments in research and in symptom-reduction-focused treatment approaches. This results in a lack of consideration of the unique psychosocial pressures an individual is currently experiencing or the potential protective factors against suicidal thoughts and/or acts [13]. There is a specific lack of psychologically focused work on the pathways to suicidal thoughts, feelings, and behaviors for people living with Bipolar Disorder [14]; this is despite the need to identify changeable psychological influences on suicide-related outcomes for intervention [15] and those factors or processes that most accurately determine who is at higher risk for suicide [2,15,16].

### The Current Review

The aim of the current scoping review was to identify the types of psychosocial factors studied in relation to the experience of suicidality amongst people living with Bipolar Disorder. For this review, we took a broad definition of ‘psychosocial’ as referring to factors which have a psychological nature, but which may be influenced by or interact with social factors (e.g., the individual’s social functioning or their social environment), as well as social factors such as social support and family environment. It should be noted that ‘psychosocial’ as a term is used broadly in the health literature and can encompass more exclusively psychological factors and/or processes (e.g., stress, hopelessness, personality), more social factors (e.g., job and work-related factors, relationships, bullying), as well as an interaction of these psychological and social factors [17,18,19] or a possible mediation of the effect of external social factors on outcomes through psychological understanding [20]. Whilst we took a broad approach to defining ‘psychosocial’, those factors clearly relating to symptoms or clinical risk factors for suicide (e.g., depression, substance abuse diagnoses) were not counted as ‘psychosocial’ for the purposes of this review.

Based on an initial literature search, and the completion of the full systematic searches and screening processes, it was apparent that the literature was heterogenous and large in terms of the number of returned studies. Therefore, a scoping review format was decided at this point to provide a clearer overview of the types of psychosocial factors that have been studied. Scoping reviews aim to provide an overview of the extent of an existing literature, regardless of its quality, are appropriate for mapping out an unknown or developing literature, and can direct future full systematic reviews and meta-analyses [21,22,23,24]. Given more recent attempts to develop precise, testable models of the experiences and ‘psychosocial’ processes implicated in suicidal thoughts and acts [15,25,26,27,28], a scoping review format was deemed appropriate to identify the types of psychosocial factors studied in relation to suicidality and Bipolar Disorder.

## 2. Materials and Methods

### 2.1. Search Strategy

The present review was conducted in line with best practice recommendations for scoping reviews [23,24,29,30,31]. Literature searches were conducted on four databases in March 2022 (Medline, PsycINFO, Scopus, and Web of Science) using two broad set of search terms relating to (1) Bipolar Disorder, and (2) Suicidality (inclusive of non-suicidal self-injury, NSSI). Top-up searches using the same databases, search terms, and eligibility criteria were conducted in November to identify literature published from March 2022 until the end of October 2023.

Based on an initial literature searching exercise, and to ensure the maximum inclusivity of the searches, we included more specific search terms relating to suicide and self-harm to account for a range of self-injurious behaviors which may or may not be motivated by suicidal intent. Broad search terms relating to Bipolar Disorder (based on different symptoms and mood episodes) were also used to ensure maximum inclusivity. Given the substantial number of possible psychosocial factors, and the inconsistent use of this term in the literature, we did not run searches with a ‘psychosocial’ set of search terms and instead screened titles/abstracts and full texts for psychosocial factors to ensure maximum inclusivity. In addition, no restriction on sample or participant age was applied to the searches. An example (full) search string for the Medline database is provided below:

MH (mania or manic or manic episode or hypomania or manic depression or manic depressive disorder or bipolar or bipolar i or bipolar ii or bipolar disorder or bipolar affective disorder or bipolar depression) AND MH (suicid* or suicidal ideation or suicidality or suicide attempt or self injury or deliberate self harm or self mutilation or self injurious behavior or self-destructive actions or suicidal)

The initial searches were conducted from the first indexed timepoint of the database until the end of February 2022 and limited to English-language articles and peer-reviewed articles. Top-up searches were conducted for the period between 1 March 2022 and 31 October 2023. The review was registered on the Open Science Framework (the registration and review documents can be accessed via https://osf.io/276sh/).

### 2.2. Eligibility Criteria

Articles were eligible for inclusion in the current review if:

1. They constituted an original piece of empirical research (any qualitative, quantitative, or mixed methods, excluding literature reviews and commentaries).
2. The article was peer reviewed.
3. The study sample consisted of people with lived experience of Bipolar Spectrum Disorder (diagnosis confirmed by a researcher using a structured clinical interview, verified by clinician, or self-reported).
4. The study focused on psychosocial etiology, process, or related factor implicated in the experience of suicidality for people living with Bipolar Disorder (as discussed in ‘The Current Review’ section above).
5. The study reported any form of suicidality-related outcome (i.e., suicidal ideation, other suicide-related thoughts or feelings, urges, plans, attempts, behaviors), including non-suicidal self-injury or self-harm.
6. The article was available in the English language.

In terms of exclusion criteria, articles were ineligible for the review if they used a mixed clinical/transdiagnostic sample but did not report data specific to people with a Bipolar Disorder diagnosis (i.e., did not stratify analyses by group). Conference presentations, letters to the editor, and student dissertations and theses were also excluded from the present review.

### 2.3. Screening and Selection

**Figure 1:**
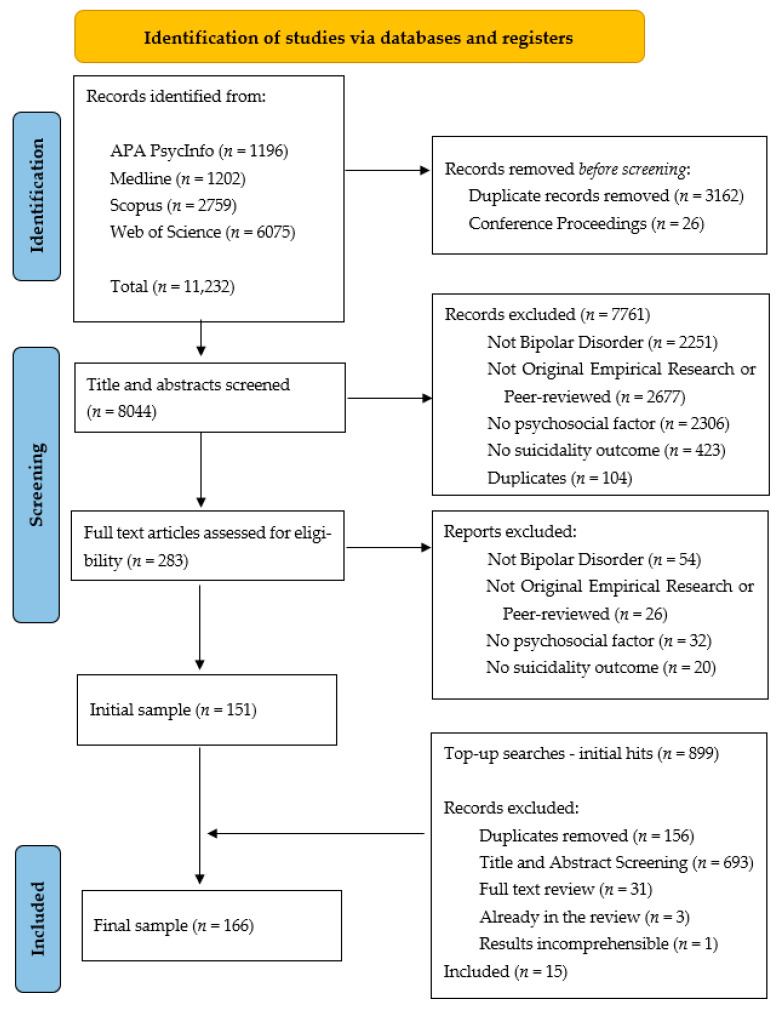
PRISMA Flowchart detailing the selection and screenings of sources for the present scoping review.

Rayyan.ai, an online web application specifically designed for managing systematic literature reviews, was used to organize the initial screening stages [32], with Covidence used to organize the top-up searches and screenings [33]. After removal of the duplicated hits (see Figure 1 for the PRISMA Flowchart [34]), two authors (RD, AD) screened the titles and abstracts of the remaining articles for eligibility based on the criteria outlined in Section 2.2, and second rated the other reviewer’s screenings. Disagreements in screening eligibility were discussed between the two authors, and a third author independently reviewed a small sample of these articles (10%), with the final eligibility agreed through discussions. Disagreements in screening mainly related to the definition and operationalization of ‘psychosocial causes’ in the reviewed literature, typically due to ambiguity in the source article’s definition and description of measures used.

### 2.4. Data Extraction

A data extraction spreadsheet was used to collate key study information from the full text reading of the identified literature. Information was extracted about the study samples (i.e., size, type such as community-based or hospital-based samples), how diagnoses of Bipolar Disorder were determined, details of study designs and methodologies, whether studies were based on an explicit theory of suicidality or self-harm, the psychosocial factors and outcomes studied, and the relationships reported between these psychosocial factors and outcomes (e.g., directions of any significant relationships observed, non-significant findings).

### 2.5. Data Synthesis

Due to the volume and heterogeneity of the identified studies, we present the reviewed articles in a narrative format structured by the type of psychosocial factors studied, a frequency count of the factors investigated, a brief overview of the assessments used to measure these factors, and a brief selective overview of the literature’s key findings.

## 3. Results

### 3.1. Overview of Results

Figure 1 details the PRISMA Flowchart and screenings of the studies included in the review.

A sample of 166 published studies was initially identified for inclusion (*n* = 151 from the initial searches plus *n* = 15 studies from the top-up searches in November 2023). Four articles reported analyses from two larger studies and featured similar analyses, assessments, and sample characteristics, and so these four articles were treated as two studies in the synthesis [14,35,36,37] (making a sample of 164 studies). Only one study in the final sample was qualitative in design [38] and featured one-to-one interviews focusing on participants’ experiences of Bipolar Disorder and the social factors influencing their past experiences of suicidality. The remaining 163 studies were all quantitative by design; most of them were cross-sectional and were primarily focused on participant- and/or clinician-reported measures of symptoms and psychosocial factors (*n* = 143), with 18 prospective studies (ranging from shorter-term follow-ups of approximately 3–4 months, e.g., [37,39], to longer-term follow-ups of 6–8 years on average [40,41]), and a further two studies which analyzed hospital records and patient chart data [42,43].

Most of the reviewed studies (*n* = 156) were not explicitly based on a theory of suicidality or NSSI in terms of their aims, research questions, or hypotheses. Three quantitative studies explicitly tested a single model of suicidality, including the Bipolar Suicidality Model [2,44] and the Interpersonal Theory of Suicide (IPTS) [27,45], with two articles [14,37] from the same larger study testing hypotheses based on the IPTS and other models (the Schematic Appraisals Model of Suicide, SAMS [26], and the Cry of Pain model, CoP [46]). In addition, Kapoor et al. (2023) [47] based their hypotheses on the IPTS and Shneidman’s concept of psychache, a form of psychological pain associated with suicidality [48]. A further study by Lamis and colleagues [49] investigated the potential risk and protective factors for suicidal ideation, partially inspired by the IPTS (e.g., childhood sexual abuse, religious and existential well-being), amongst adults with Bipolar Disorder. The interview schedule used in Owen and colleagues’ qualitative study (2015) [38] was partially informed by existing ideation-to-enaction models of suicidality, including the IPTS, SAMS, and CoP models, as well as the Integrated Motivational–Volitional Model of Suicide [25,50]. Hence, most of the reviewed literature had little basis in theories of suicidality or self-harm/NSSI.

### 3.2. Types of Psychosocial Factors Studied in Relation to Bipolar Disorder and Suicidality

Table 1, below, lists the identified psychosocial factors implicated in suicidality for people with Bipolar Disorder in the reviewed studies. The most studied factors were trauma (inclusive of childhood traumas, early adversity, and adult lifetime traumas), life stress (inclusive of stressful life events and general stress levels), impulsivity (primarily measured using the Barratt Impulsivity Scale [51]), social support and relationship-related factors (excluding family relationships), hopelessness, and personality and temperament.

#### 3.2.1. Trauma and Stressful Life Events

The most widely studied psychosocial aetiology was the experience of past traumas and/or stressful life events (*n* = 66). The former were typically early adverse experiences such as childhood emotional, physical, and/or sexual abuse or neglect.

Most studies that focused on trauma specifically used a participant self-report measure to assess past experiences of trauma, including the Childhood Trauma Questionnaire (CTQ, *n* = 31, [47,49,52,53,54,55,56,57,58,59,60,61,62,63,64,65,66,67,68,69,70,71,72,73,74,75,76,77,78,79]), the Early Trauma Inventory (*n* = 4, [80,81,82,83]), and other validated self-report measures (*n* = 5, [40,84,85,86,87]), or used novel but unvalidated items or checklists (*n* = 4, [88,89,90,91]). Other studies assessed histories of trauma via chart and medical record review [42,43,92] or from medical questionnaires or clinical interviews (*n* = 8, [92,93,94,95,96,97,98,99]; note one study used both medical records and clinical interviews to assess trauma [92]).

A history of trauma, typically measured as an overall score or a total count of the number of traumas experienced, was generally associated with worse suicidality outcomes, including more severe ideation (e.g., [40,80,82]), a history of suicide attempts (e.g., [52,57,66,69,84,85,98]), and a history of self-harm/NSSI [75,86]. That said, two studies reported no significant differences or associations between histories of suicide attempts and past exposure to trauma [61,97].

There was evidence that more specific forms of trauma and early adversity were associated with worse suicidality, but this varied across studies. For example, studies reported associations between suicidal ideation and childhood sexual abuse [49,94] or emotional abuse with ideation [100], with childhood emotional abuse, sexual abuse, and general trauma being associated with suicidal ideation in general and with passive suicidal ideation [80]. On the other hand, there was evidence of similar associations between suicidal ideation and all the commonly recognized form of childhood traumas [68] (physical, sexual, and emotional abuse; and physical and emotional neglect). Grillault Laroche and colleagues (2022) reported similar associations between the CTQ subscales with suicide attempts, but their network analysis indicated that suicide attempts were more strongly associated with a ‘community’ of physical, emotional, and sexual abuse [63].

Non-traumatic albeit stressful life events included negative life events (e.g., losing employment, bereavement) and those that were not necessarily negative but still considered stressors given the significant life changes (e.g., moving house, marriage). Traumas and broader life stress-related experiences were combined in this review because several studies included measures which had conceptual overlap (i.e., they did not clearly separate or parse traumatic versus non-traumatic events) [81,84,101,102,103]. The two studies using the St Paul-Ramsey Scale of stressful life events found no relation to suicide attempts [101,102], or suicidal ideation [102], whereas the others found an association between life events and suicide attempts [81,103]. In a further three studies, it was unclear if the measure of life stressors or life events included trauma or not [42,104,105], but there were significant associations with suicide-related outcomes in two of these [104,105]. Of the seven studies that focused on stressful life events, four used the Social Adjustment Scale (or a variant of this). In all of these, a history of stressful life events was associated with suicide-related outcomes [106,107,108,109]; in one, this relationship was moderated by a candidate risk gene [106]. The final three studies used various life events checklists, and all had significant associations with suicide-related outcomes [110,111,112].

There was inconsistency in the literature in the specificity of assessments of trauma and life stressors. There was also a tendency to only measure the total number of traumas experienced, with a lack of assessment of the severity and duration of trauma or the source or perpetrator of the trauma (e.g., in relation to abuse), and no focus on the subjective experience or sense-making of trauma-related experiences (i.e., no qualitative studies).

#### 3.2.2. Impulsivity

Impulsivity was the second most widely studied psychosocial aetiology (*n* = 40). Most studies assessed impulsivity using a form of the Barratt Impulsivity Scale [113] (BIS, *n* = 33, [61,64,84,86,101,102,114,115,116,117,118,119,120,121,122,123,124,125,126,127,128,129,130,131,132,133,134,135,136,137,138,139]). Other studies used alternative self-report measures of impulsivity and related constructs, including assessments of positive and/or negative urgency [86,137,140], impulsive aggression (e.g., the Impulsive/Premeditated Aggression Scales [141]) [73,137], sensation seeking [142], impulsive traits associated with Borderline personalities [99], impulsive temperaments [135], and single-item measures of momentary impulsivity [39]. Four studies featured experimental assessments of impulsive responding and behaviors, such as the Iowa Gambling Task (IGT) and other continuous performance tasks [136,140,143,144].

Greater impulsivity was typically associated with worse suicidality (based on measures of higher self-reported trait impulsivity), including with more severe suicidal ideation [86] and a history of suicide attempts (e.g., [84,101,114,115,117,128,131,137]). Tendencies to act impulsivity when experiencing strong emotions (i.e., positive and negative urgency) had differential associations with outcomes, with both positive and negative urgency correlated with suicidal ideation, but only positive urgency positively was associated with a history of self-harm/NSSI or suicide attempts [86]. Few studies investigated indirect relationships involving impulsivity and other aetiologies, although there was evidence that impulsivity mediated the relationship between childhood abuse and suicidal behavior [84], suggesting a possible developmental pathway to suicidality in Bipolar Disorder. One further study indicated higher BIS scores for motor impulsivity, attentional-cognitive, and non-planned impulsivity for participants with histories of suicide attempts versus those with ideation histories without attempts [120].

There was evidence that such impulsivity–suicidality associations did not hold in multivariate models when other clinical factors were included (e.g., substance use behaviors and diagnoses) [86,127]. Some studies reported null findings, with impulsivity not associated with suicidal ideation [122,133] or a history of suicide attempts [102,116,118,129] (including when impulsivity was assessed behaviorally using the IGT, [140]), and no differences in impulsivity in people with histories of one versus multiple suicide attempts [125].

Impulsivity appeared to have inconsistent relationships with suicidality outcomes depending on the nature of impulsivity assessed (trait versus state), the measurements used (self-report versus behavioral tasks), and suicidality outcomes (e.g., ideation versus behaviors), with some overlap between impulsivity and other related factors also noted (e.g., substance use, aggression [130]). Despite these relationships, there was some suggestion that impulsivity may not be a reliable indicator of suicide risk for people with Bipolar Disorder [134].

#### 3.2.3. Social Factors

Twenty-three studies focused on factors relating to (overall) social functioning [65,89,145,146,147], perceptions of social support [14,37,38,68,148,149,150,151], and the quality of interpersonal relationships [41,94,99,152,153,154,155], as well as occupational and school functioning (e.g., workplace office, school-based bullying) [145,154,156]. It should be noted that some studies focused on more than one social factor. Family-specific factors are discussed separately below.

Poorer social support was generally associated with worse suicidality, including increased ideation [37,68] and a history of suicide attempts [148]. Although, one study found no direct relationship between perceived social support and suicidal ideation at a four-month follow-up, but did find indirect relationships via changes in hopelessness, defeat, and entrapment [14]. Poorer relationship qualities were similarly related to worse ideation [41,94] and histories of suicidal behaviors [154]. Poorer social functioning (relating to irregular social activities) was associated with current passive and active suicidal ideation and suicidal plans [147], with poorer occupational functioning similarly associated with greater suicidal behavior [154]. Avoidant relationship styles were more common amongst participants with more severe/repeated histories of suicide attempts compared to those with a prior attempt, lifetime ideation only, and no lifetime suicidal behavior [153]. Adolescents with Bipolar Disorder and a history of either suicidal ideation or suicidal behavior reported being bullied more than participants with histories of NSSI-only [89]. Adults with histories of being bullied were more likely to have histories of suicide plans and suicide attempts, inclusive of cyberbullying experiences [157] and past experiences of childhood bullying [76].

There were non-significant relationships reported between suicidality and social factors in relation to (overall) social functioning [146], bullying victimization [152], interpersonal problems [99], social adjustment [156], social networks [155], or social support [149,150,151], and no relationship between current ideation or suicide attempt histories and social functioning (when adjusting for clinical co-variates [65,145]). Finally, the only qualitative study in the review, by Owen and colleagues [38], identified a range of social factors which people with Bipolar Disorder associated with worsening (e.g., low perceived social support, negative social events) or protecting against (e.g., reflecting on and recalling past positive social experiences) suicidality. Aside from this final study [38], there was little focus on identifying the social factors that have a protective or buffering effect against suicidality.

#### 3.2.4. Hopelessness

Twenty studies included self-report measures of hopelessness; 18 used the Beck Hopelessness Scale [158] (BHS, [14,37,82,101,102,109,110,133,147,150,151,159,160,161,162,163,164,165]) and two studies [166,167] used the Hopelessness Scale for Children which was derived from the BHS [168]. Greater hopelessness was associated with worse suicidality across studies, including worse suicidal ideation [14,37,133,151,159,165,166,167], a history of suicidal behaviors/attempts [102,150,151,159,161,163], a higher suicide risk [163], and higher odds for future suicidal behavior and death by suicide [150,162]. Although, some studies reported no significant relationships between hopelessness and suicidality in multivariate models alongside other clinical variables (e.g., depressive symptoms) [101,160,164].

There was some evidence of associations between hopelessness and more specific aspects of suicidal ideation, with total BHS scores and subscale scores (i.e., feelings about the future, future expectations, and loss of motivation) associated with both passive and active forms of suicidal ideation, but more hopeless expectations of the future were only associated with suicide plans [82]. Another study reported that BHS ‘feelings about the future’ scores were associated with passive and active ideation and suicidal plans, but BHS total scores and the other subscales were only associated with passive ideation [147]. Hopelessness (total BHS scores) mediated the relationship between depression and passive ideation, with the emotional component of hopelessness also mediating the relationship between social rhythm disturbance (irregular social activities and behaviors) and active ideation with suicidal plans [147]. There were also some instances of hopelessness becoming a non-significant predictor of suicidality outcomes when other psychosocial aetiologies and covariates were added to multivariate analyses (e.g., [82,147]).

#### 3.2.5. Personality and Temperament

Nineteen studies assessed some form of personality or temperament, including measures of the five-factor trait model of personality using a form of the NEO-PI or NEO-FFI [62,110,160,162,169], a short form of the Eysenck Personality Questionnaire [170], or a measure of maladaptive personality traits [171] (e.g., the SNAP-2 [172]). Other studies assessed more bipolar-/mood-related temperaments using a version of Cloninger’s TCI [119,134,135,173,174,175], the TEMPS scale [120,139,161,176], or an interview-based assessment of temperament that preceded the TEMPS scale [177], or they did not clearly specify the measure used [104].

In terms of temperament, a history of suicide attempts was associated with lower self-directedness and higher self-transcendence [175], but other studies reported higher scores in harm avoidance and reward dependency amongst those with a history of suicide attempts [173]. Other studies reported more cyclothymic [104] or depressive temperaments [161,177] amongst those with histories of suicidal behaviors, although such associations were not always consistent with reports of higher cyclothymic, hyperthymic, and/or irritable temperaments for those without histories of suicidal behavior versus those with such histories [161,176,177]. Higher scores on a measure of suicide-proneness personality traits (capturing thoughts and behaviors relating to self-harm and suicide) were associated with more severe suicidal ideation and more time spent with ideation at a 1.5-year follow-up in young adults with Bipolar Disorder [171]. There were also reports of no difference in temperament between individuals with and individuals without histories of suicidal behaviors [134] or between participants with histories of suicidal ideation (no behaviors) and those with histories of both ideation and behaviors [120].

Framed in terms of more traditional trait-based approaches to personality, suicidal ideation was associated with higher Neuroticism amongst people with Bipolar Disorder without a history of suicidal behavior, and higher Openness and lower Extraversion amongst those with a history of suicide attempts [160]; however, one study reported higher Neuroticism amongst those with a history of suicide attempts [110]. Higher Neuroticism scores were also associated with higher odds for new-onset suicidality (ideation and/or behavior) over an 18-month follow-up [169]. There was some indication of differences in personality/temperament according to different manifestations of suicidality, in that Neuroticism was implicated in suicidal thoughts rather than behaviors/attempts.

#### 3.2.6. Irritability and Aggression

Eighteen studies measured aspects of irritability and/or aggression, including anger and hostility, as predictors of suicidality outcomes. All studies used some form of self-report measure, namely, the Buss–Durkee Hostility Inventory [101,102,125,126,128,130,133,155], the Buss–Perry Aggression Questionnaire [55,61,109,139], the Brown–Goodwin Aggression Inventory [101,102,129,130,155], and the Impulsive/Premeditated Aggression Scale [73,137], alongside other validated measures of aggression, irritability, and hostility [56,133,166,178].

Generally, increased irritability and aggression were associated with worse suicide outcomes, including a history of suicide attempts/behaviors [101,102,126,130,137,155] and greater suicidality risk [73]. One study reported significant differences in verbal aggression between those with and without a history of attempted suicide, but no differences on the anger or physical aggression subscales on the Buss–Perry Aggression Questionnaire [109]. Adolescents with Bipolar Disorder and current suicidal ideation reported greater difficulties regulating anger compared to those without ideation [166]. Hostility was also associated with a history of suicide attempts/behaviors [102,109,128,130], particularly amongst people who were more irritable and who displayed indirect hostility towards others (e.g., gossip) [128]. There were also significant relationships between irritability and hostility and suicidal ideation [133]. Impulsive aggression also acted as a mediator of the relationship between childhood sexual and emotional abuse on suicidality [73], and there was evidence of associations between impulsivity and hostility amongst people with Bipolar Disorder and a history of suicide attempts but not for those without a history of attempts [126]. A cluster analysis of measures of temperaments and aggressive behavior identified a self-aggression cluster that was associated with significantly higher rates of lifetime suicide attempts compared to clusters characterized by aggression towards others and non-aggressive behaviors [178].

Other studies reported no significant difference between those with and those without a history of suicide attempts in regard to aggression [61,129], past acts or feelings of violence [109], or lifetime/trait hostility [101,125]. One study reported lifetime aggression was significantly higher in those with a history of attempts, with no differences for lifetime hostility [101]. There was some inconsistency across studies in how hostility and aggression were operationalized. Anger/aggression and hostility may represent similar yet different constructs, and there might be subtle differences between more aggressive behaviors and a more cognitive/schema-based hostility [179]. It was notable that aggression, hostility and irritability in the reviewed studies were only measured by self-report, often based on total scores on measures rather than analyzing by specific subscales (e.g., [155]). There was also little consideration of contextual aspects of these factors, and a focus on lifetime experiences or traits rather than more dynamic, transient, or in-the-moment experiences.

#### 3.2.7. Quality of Life

Fifteen studies featured measurements of Quality of Life (QoL) across various life domains, including the World Health Organization’s QoL Instrument Short Version (WHOQOL-BREF [180]) [111,145,149,157,181,182], the Quality of Life Enjoyment and Satisfaction scale (QLES [183]) [93,105,169], child-appropriate QoL measures (e.g., the KINDL-R [184]) [185,186], and a specific QoL scale for Bipolar Disorder [146], or they used other validated QoL scales [35,36,187]. There was typically a negative relationship between QoL and life satisfaction and suicidality, including with suicidal ideation [35,36,93,146,157] or a history of suicide attempts [110,145,181,182,185]. Life satisfaction (via the QLES) was also a significant mediator of the relationship between depressive symptoms and suicidal ideation [93]. In contrast, higher QoL was associated with lower odds ratios for suicide attempts [111].

Other studies reported no significant differences in QoL domains (e.g., physical health, social relationships) between participants with a history of suicide attempts/behaviors and those with no history [149], no association between QoL and suicide attempts [187], or no QoL–suicide-risk relationship [169]. QoL also did not remain significantly associated with a history of suicidal behavior in multivariate models including other clinical covariates (e.g., [145]).

#### 3.2.8. Cognitive and Neuropsychological Factors

Fourteen studies included assessments of cognitive and neuropsychological processes. A wide variety of neurocognitive assessments of executive functioning, memory, attention, and processing speed were used, for example: the Stroop task [61,188,189,190,191]; digit span tasks [189,192]; spatial working memory tasks [193]; nonverbal memory and learning tasks [194]; continuous performance tests of attention [134,136,144,190,195]; the Wisconsin Card Sorting Task [191,192,194,195]; and the Iowa Gambling Task [134,144,191,192]. As discussed earlier, studies also used neurocognitive tests to assess impulsive responding to stimuli (e.g., [143]). Other studies included subjective self-report assessments of cognitive dysfunction [189,190] and neurocognitive performance [35,36], including the Cognitive Failures Questionnaire, which assesses perceived difficulties in memory, attention, and motor functioning [196].

Numerous studies reported a neurocognitive-impairment–suicidality association, with greater impairments and more impulsive-like responding associated with worse outcomes and more severe histories of suicidal behaviors/attempts [136,143,194]. Although there was evidence of limited differences in neurocognitive performance between those with and those without histories of suicidal behaviors across tests in one study, the authors reported that histories of suicidal behaviors/attempts were associated with worse performance on a continuous performance test (capturing impulsive errors), a relationship that was not observed in regard to other tasks [134]. Impairments in visual and working memory were associated with more severe current ideation [189], with evidence of a moderation of the objective cognitive performance-to-ideation relationship by depressive symptoms in the same study. Reduced cognitive flexibility (based on slower performance on reversal tasks) was associated with longer times experiencing suicidal ideation in children and young adults with Bipolar Disorder [193]. Malloy-Diniz et al. (2009) reported significantly worse decision making (based on the Iowa Gambling Task) amongst participants with Bipolar Disorder and a history of at least one prior attempt compared to those with no history of suicide attempts (and no group differences on other test of memory, attention or executive functioning [191]); this finding was later replicated in a similar study by the same authors [144]. Martino and colleagues (2011) reported similar riskier decision making on the Wisconsin Card Sorting Task amongst participants with a history of suicide attempts compared to those with no prior attempts, but no differences on other assessments of executive functioning [192].

Studies reported no significant differences in cognitive functioning between participants with history of suicide attempts and those with no prior history of suicidal behavior [61,188], or between participants with and without recent (past year) ideation or attempts [195]. However, other studies reported that greater subjective cognitive complaints were positively associated with the number of prior suicide attempts [190] and with suicidal ideation [35,189]. Overall, there were some mixed findings for the role of neurocognitive factors in suicidality but there was some evidence of worse decision making being associated with a history of more severe suicidality/attempts.

#### 3.2.9. Coping Styles and Emotion Regulation

Fourteen studies investigated links between coping styles or emotion regulation and suicidality outcomes. Seven studies included a variety of self-report measures of coping [109,166,167,186,197,198,199], with mixed findings. Studies reported no significant coping-suicidality relationships or differences in coping styles between groups differentiated by the type or severity of suicidality [166,199] (e.g., between children/adolescents with different severities of suicidal ideation and behaviors [167]). Aksoy Poyraz et al. only observed differences in dysfunctional coping styles (higher in participants with no suicidal behaviors versus a history of suicidal behaviors) but not emotion- or problem-focused coping [197]. Similarly, worse problem-solving-focused coping but higher emotion-focused disengagement coping was found in adults with Bipolar Disorder and a lifetime history of suicide attempts versus those without a lifetime history [109]. Emotion-approach coping has also been associated with lower suicide risk (based on a composite measure of ideation/behaviors) [198]. The one longitudinal study that examined emotional coping found no associations between children’s suicidal ideation intensity and their own coping, or with their parents’ coping styles [186].

In terms of emotion regulation, several studies reported associations between greater difficulties regulating emotions as measured by the Difficulties in Emotion Regulation Scale (DERS: [200]) and the increased severity of suicidal ideation [138,201], suicide risk [138], and suicide attempts [202]. A separate study found no direct relationship between difficulties in emotion regulation and suicidal ideation, but DERS total scores did act as a mediator between disrupted social rhythms and ideation [203]. Two studies found significant associations between rumination and suicidality (risk measures based on behaviors and ideation) [204], whereas, in one longitudinal study, there was no direct association, although rumination amplified the relationship between self-criticism and ideation [44].

#### 3.2.10. Family Factors

Nine studies focused on specific family-related psychosocial factors, particularly family functioning and communication styles, in suicidality outcomes. Seven of these studies sampled children or adolescents with Bipolar diagnoses [41,94,112,167,185,186,205], with the remaining two studies sampling adults [145,206]. Four studies reported associations between poorer overall family functioning and past or current suicide attempts [145,185,206] as well as elevated probability for future suicide attempts [41]. Ellis et al. (2014) reported that, whilst family cohesion and adaptability were not associated with suicidal ideation amongst adolescents with Bipolar Disorder, families with parents high in expressed emotion (i.e., who were critical, demonstrated overinvolvement, or were hostile towards their child based on five-minute speech samples) were more likely to have children with current suicidal ideation [205].

Across other studies with youth with Bipolar Disorder, more severe suicidal ideation was associated with greater family conflict, more stressful family life events, and poorer relationship qualities with parents [41,94,112]. In terms of more positive associations, greater mobilization of family support and resources to obtain help was associated with lower suicidal ideation in children with Bipolar Disorder [186]. Most of the family-focused studies relied on self-report measures of parenting and the family environment, particularly focusing on the negative effects of these factors on suicidality outcomes.

#### 3.2.11. Cognitive Styles

Eight studies focused on some form of cognitive style not captured elsewhere in the review categories. This ‘cognitive style’ category captured a broad range of constructs typically relating to individuals’ perceptions of themselves and/or their social environment, with little overlap across studies in terms of the assessments and constructs studied. The exception to this was two studies that included measures of maladaptive schemas [207,208], which were generally higher in those with a history of suicide attempts, particularly entitlement. One study reported a moderate (cross-sectional) correlation between subjective cognitive dysfunction (as a result of the COVID-19 pandemic) and suicidal ideation [146]. In the remaining two studies, there was a general pattern of maladaptive cognitive styles (intolerance of uncertainty, metacognitive beliefs) being related to increased suicidality [153,209].

This category also includes those studies using measures of psychosocial factors that were selected based on psychological theories of suicide. These theories were broadly supported with evidence that increased suicidal ideation and histories of attempted suicide were associated with thwarted belongingness and perceived belongingness (Interpersonal-Psychological Theory) [45,47], with more severe ideation being associated with appraisals of defeat and entrapment (which are featured in various theories of suicide) [14,37] and self-criticism being associated with more severe ideation amongst those with high scores on a measure of negative cognitive styles (in line with the Bipolar Suicidality Model) [44].

#### 3.2.12. Insight

Five studies included a self-report assessment of ‘insight’, broadly defined as an awareness of Bipolar Disorder as an experience and its associated symptoms, with studies using the Insight Scale for Affective Disorders [122,210,211], the Schedule for Assessment of Insight [195], or shortened versions of existing insight scales [159]. There were mixed findings across studies, with greater insight associated with worse suicidal ideation [159] and attempts [211], but some evidence of no significant differences in insight between participants with and without current ideation, but evidence that histories of suicide attempts are associated with worse insight [210]. Two studies reported significant relationships between greater insight and greater suicidal ideation and/or more attempts [122,195]. Insight, in these studies, was limited to the individual’s level of insight/awareness of their own experience of Bipolar Disorder (e.g., as total ‘insight’ scores).

#### 3.2.13. Reasons for Living

Five studies [61,101,102,130,155] used Linehan et al.’s Reasons for Living Inventory (RfLI) [212]. There were mixed findings, with some studies reporting no relationships between Reasons for Living scores and suicidality or no difference in scores between groups of participants with Bipolar Disorder with and groups without histories of attempted suicide [61,101,130]. Two studies reported significant associations between RfLI scores and suicidality, with an increased likelihood of suicidal behavior associated with lower moral/religious objections to suicide [155], and a history of suicide attempts associated with a lower (total) RfLI score when controlling for current levels of depression [102].

#### 3.2.14. Resilience

Six studies included an explicit measure of resilience (all self-report assessments), including the Connor–Davidson Resilience Scale [213,214], the Resilience Scale for Adolescents [123], and the Resilience Scale for Adults [83,139,201]. One study reported no significant association between resilience and history of suicide attempts [213]. Other studies reported generally lower psychological resilience in people with histories of suicide attempts versus those without a history of prior attempts [123,139,214]. Şenormancı and colleagues (2020) noted some differences between attempter status groups depending on the Resilience Scale for Adults subscales, with higher perception-of-the-future scores associated with less severe histories of past suicide attempts, but family cohesion scores higher in participants with at least one prior suicide attempt versus those with no history [139]. Palagini et al. (2022) reported positive relationships between passive, active, and total suicidal ideation scores and low (total) resilience, with suicide plans positively associated with low resilience in terms of future planning [83]. A separate study by the same authors also reported similar associations between low overall resilience and low resilience in terms of future planning and suicidal ideation [201]. Generally, greater psychological resilience was associated with better outcomes, although all these studies were cross-sectional by design.

#### 3.2.15. Other Factors

Other psychosocial factors featured in the reviewed studies (all based on self-report assessments) included religiosity and spirituality [49,115,199], self-esteem/self-concept [156,166,167,182,186], perceived stigma [182], locus of control [166], identity confusion [99], time perspective [215], psychache [47], and the fear of COVID-19 [146]. Religiosity had mixed associations with past histories of suicide (no relationship in one study [199]; intrinsic religiosity was associated with lower odds for attempts in one study [115]), with another study suggesting stronger protective effects of existential wellbeing (e.g., life purpose) against suicidal ideation versus religiosity [49]. Three studies, all with children and/or adolescents with Bipolar diagnoses, reported lower self-esteem/self-concept scores were associated with stronger suicidal ideation [166,167,186], with lower self-esteem also reported amongst adults with Bipolar Disorder and a history of suicidality (past ‘serious’ suicidal thoughts and/or at least one prior suicide attempt) [156].

One study reported that suicidal ideation was associated with tendencies towards past negative time perspectives and future time perspectives, indicative of a preoccupation with the past and possibly hopelessness about the future amongst those who reported more intense current ideation [215]. Participants who scored higher on a self-report measure of psychache (a form of psychological pain) were more likely to be classified as current suicide ideators in a discriminant analysis [47]. Türk and Uğurlu (2023) reported that participants with Bipolar Disorder and a history of attempted suicide had lower self-esteem and higher perceived stigma compared to those without histories of attempts [182]. The remaining studies reported no significant association between a fear of COVID-19 and suicidal ideation [146], that a more external locus of control was associated with greater suicidal ideation amongst adolescents with Bipolar Disorder [166], and that greater identity confusion was found in adolescents and young adults with Bipolar Disorder histories of suicide attempts or NSSI compared to those with no suicidality/NSSI history [99].

## 4. Discussion

Given the developing nature of the research literature, this scoping review aimed to identify the key psychosocial factors implicated in the experience of suicidality amongst people with lived experience of Bipolar Disorder.

### 4.1. Key Findings

The most studied factors associated with experiences of suicidality for people living with Bipolar Disorder were traumatic (i.e., abuse and neglect) and stressful life events, impulsivity, social functioning, personality and temperament, hopelessness, and factors relating to aggression and impulsivity, amongst others (see Table 1). The literature was primarily quantitative and was heavily reliant on self-report assessments of various aetiologies in primarily cross-sectionally designed studies.

The reviewed literature also varied significantly in terms of the types of factors studied at the same time/in the same study, which seemed indicative of the general lack of theory-based research questions. The limited theoretical basis to this literature poses a challenge for understanding the pathways to suicidality experienced by people living with Bipolar Disorder, particularly in identifying and testing the causal factors implicated in suicidality, in further refining theories, and in identifying changeable factors for intervention.

The literature also tended to focus on factors associated with negative outcomes, particularly relating to negative life experiences (e.g., trauma, stress, early adversity, difficult family environments) and impairments (e.g., in neurocognitive processes, emotion regulation). Focusing on more positive or protective factors which buffer against suicidality was not common, despite the potential for developing effective interventions [216]. There were also examples of studies focusing on possible protective factors from a deficit perspective, such as the relationship between low psychological resilience and suicidal ideation [83]. Although we have identified significant literature focusing on the role of psychosocial causes implicated in suicidality for people living with Bipolar Disorder, there is a lack of focus on protective or buffering factors against suicidality.

### 4.2. Evaluation of the Reviewed Literature and Recommendations for Future Research

Due to the scoping nature of the review, we cannot draw definite conclusions regarding the quality of the literature. There were, however, several observations made during the literature screening and reviewing, including that most studies focused on adult samples, with few studies sampling children and adolescents with diagnoses of Bipolar Disorder. There was also some lack of clarity in the reviewed studies regarding the confirmation of diagnostic status, in terms of who conducted and verified diagnoses, or which tools were used to confirm diagnosis. Although, some studies did report corroborating diagnoses with medical records (e.g., [39,188]), via consensus or review by clinicians (e.g., [101,112]), or by conducting independent rater checks of diagnostic interviews (e.g., [75,86,151]).

We also observed a significant variation in the assessments used within each psychosocial factor category identified in the review, with variation across studies in terms of the specificity of these assessments (e.g., between broader assessments based on total scores to the analysis of individual subscales/types of psychosocial factors). This was especially notable in the trauma-focused studies, where there was variation between studies looking at a broader count of traumas experienced (a frequency or total score, e.g., [40,65,66]) to finer-grained analyses indicating the unique influences of specific traumas on suicidality (e.g., [63,71,100]). Studies also tended to focus on relatively straightforward direct or main effects, rather than exploring more complex or conditional relationships between psychosocial factors and suicidal experiences. That said, there were examples of studies investigating indirect and interaction effects (mediations, moderations, e.g., [37,84,93]) and using network analysis [63] and structural equation modelling [100,106].

There was variation in how ‘suicidality’ was defined and assessed in the literature, including in the use of validated assessments, the specificity of the outcomes and the timeframes assessed. Studies differed in whether they measured lifetime ideation or behaviors according to a binary outcome of presence versus absence or used more specific and nuanced assessments distinguishing between different forms of suicidal thoughts (such as passive versus active ideation, and suicidal ‘desire’, e.g., [80,82]). There were also instances of studies combining ideation and behavior into a single suicidality or ‘suicide risk’ construct (e.g., [156,169,204]). This lack of specificity, and the combining of ideation and behaviors into one construct, is problematic given that some variables might be stronger predictors of thoughts than behaviors and that experiencing suicidal thoughts and engaging in non-suicidal self-injury does not always translate into future suicidal behaviors [217,218]. Based on the present scoping review, there appears to be a lack of consideration of these issues in the context of the lived experience of Bipolar Disorder.

The largely cross-sectional (quantitative) nature of the literature, and the reliance on memory recall and retrospective assessments of precursors to suicide (e.g., early adversity and childhood traumas), as well as past experiences of suicidality, poses limits on the understanding of how certain factors are associated with suicidality over time, especially over the lifespan [38]. There is a particular lack of understanding of the dynamic nature of factors that may increase or decrease suicidal thoughts, feelings, and/or behaviors at different times. The use of more prospective designs and methodologies, such as ecological momentary assessment (EMA) and network analysis, would address this gap in knowledge. Only one of the reviewed studies, however, reported using EMA and focused on assessing changes in subjective impulsivity in relation to suicidality over an 11-week period [39]. Considering the dynamic nature of suicidal thoughts, feelings, and behaviors, as well as the cyclical nature of Bipolar Disorder, there needs to be a greater consideration of the temporal fluctuations in both psychosocial factors and suicidal experiences. Indeed, there is evidence that the presence and severity of suicidal ideation and behaviors can be markedly different according to current Bipolar mood states and episodes [165].

### 4.3. Strengths and Limitations of the Present Review

As the review took a scoping format, it is not appropriate to draw conclusions about which psychosocial factors are the strongest predictors of suicidality outcomes or to extensively comment on the quality of the reviewed literature. Understanding the strength of the associations between the psychosocial factors discussed here with suicidality outcomes, and the quality of the evidence base would require a narrower, more selective, review of the literature subject to quality assessments and meta-analyses or meta-syntheses beyond the aims of typical scoping reviews [23,219]. This scoping review also cannot comment on the potential confounding variables implicated in the psychosocial factor-suicidality relationship; there may be a number of confounds or interacting variables that would require analysis through a full systematic review and meta-analysis.

This review has, however, identified a range of literature focusing on psychosocial factors associated with suicidality for people living with Bipolar Disorder, which may assist in directing future reviews and research. A key strength is that a series of comprehensive literature searches using broad search terms were conducted, subject to thorough rater consistency checks, with a significant body of literature screened and summarized. There were limitations with the search approach in that the grey and non-peer-reviewed literature was not searched and the review is broad in scope, particularly across sample/participant ages. More focused comprehensive literature reviews for children and adolescents, and for adults, may be required.

Whilst we identified a range of psychosocial factors implicated in suicidality, some of these factors may overlap with more traditional clinical risk factor approaches (e.g., trauma). We used broad search terms in the present review and relied on title, abstract, and full-text screenings, to ensure the maximum inclusivity of psychosocial factors; however, the literature still lacked a clear or consistent definition of the term ‘psychosocial’. Given the inconsistent definition of what constitutes a ‘psychosocial’ factor, it may be that other factors not featured in the present review remain important to investigate. In sum, this scoping review provides a clear summary and overview of a complex and often inconsistent literature, but it is limited in terms of providing a critical account of the strength of evidence for the identified psychosocial factors and mechanisms implicated in suicidality-related experiences for people living with Bipolar Disorder.

## 5. Conclusions

The present scoping review has identified several key psychosocial factors implicated in the experience of suicidality amongst people living with Bipolar Disorder. The experience of early traumas and life stress, impulsivity, challenges in social functioning and relationships, personality and temperament factors (especially mood-related), hopelessness, irritability and aggression, as well as dysregulation or impairments in a number of neurocognitive processes, all appear to be associated with worse suicidality related outcomes. The literature, however, is largely quantitative and cross-sectional, lacks a basis in theoretical models of suicide and/or Bipolar Disorder, and is associated with inconsistencies in how psychosocial factors and suicidality outcomes are operationalized, assessed, and analyzed. There is a clear need for more theory-based, prospective, and micro-longitudinal studies of the psychosocial factors most implicated in suicidality for people living with Bipolar Disorder, alongside more qualitative and co-produced research focusing on lived experience.

## Figures and Tables

**Table 1:** Types and frequency of psychosocial factors investigated in relation to Bipolar Disorder and Suicidality in the reviewed studies.

Psychosocial Factor	Number of Studies ^1^
Trauma and Stressful Life Events	66
Impulsivity	40
Social Factors	23
Hopelessness	20
Personality and Temperament	19
Irritability and Aggression	18
Quality of Life	15
Cognitive and Neuropsychological Factors	14
Coping Styles and Emotion Regulation	14
Cognitive Styles	10
Family Factors	9
Resilience	6
Insight	5
Reasons for Living	5
Other Factors	12

^1^ Note that some studies featured more than one psychosocial factor. Please see the OSF files for a further breakdown of the psychosocial factors measured in the reviewed studies.

## Data Availability

The review was registered on the Open Science Framework (the registration, review data, PRISMA-Scoping Review checklist, and other documents can be accessed via https://osf.io/276sh/).

## References

[1] Jamison K.R. (2000). Suicide and Bipolar Disorder. J. Clin. Psychiatry.

[2] Malhi G.S., Outhred T., Das P., Morris G., Hamilton A., Mannie Z. (2018). Modeling Suicide in Bipolar Disorders. Bipolar Disord..

[3] Pompili M., Gonda X., Serafini G., Innamorati M., Sher L., Amore M., Rihmer Z., Girardi P. (2013). Epidemiology of Suicide in Bipolar Disorders: A Systematic Review of the Literature. Bipolar Disord..

[4] Plans L., Barrot C., Nieto E., Rios J., Schulze T.G., Papiol S., Mitjans M., Vieta E., Benabarre A. (2019). Association between Completed Suicide and Bipolar Disorder: A Systematic Review of the Literature. J. Affect. Disord..

[5] Dome P., Rihmer Z., Gonda X. (2019). Suicide Risk in Bipolar Disorder: A Brief Review. Medicina.

[6] Schaffer A., Isometsä E.T., Tondo L., Moreno D.H., Sinyor M., Lars Vedel K., Turecki G., Weizman A., Azorin J.M., Ha K. (2015). Epidemiology, Neurobiology and Pharmacological Interventions Related to Suicide Deaths and Suicide Attempts in Bipolar Disorder: Part I of a Report of the International Society for Bipolar Disorders Task Force on Suicide in Bipolar Disorder. Aust. N. Z. J. Psychiatry.

[7] Turecki G., Brent D.A., Gunnell D., O’Connor R.C., Oquendo M.A., Pirkis J., Stanley B.H. (2019). Suicide and Suicide Risk. Nat. Rev. Dis. Primers.

[8] Tondo L., Vázquez G.H., Baldessarini R.J. (2021). Prevention of Suicidal Behavior in Bipolar Disorder. Bipolar Disord..

[9] Yoldi-Negrete M., Fresán-Orellana A., Jiménez-Tirado M., Martínez-Camarillo S., Palacios-Cruz L., Vieta E., Ortega-Ortiz H., Becerra-Palars C., Gutiérrez-Mora D., Camarena Medellín B. (2021). Ten-Year Course of Treated Bipolar I Disorder: The Role of Polarity at Onset. Brain Behav..

[10] Franklin J.C., Ribeiro J.D., Fox K.R., Bentley K.H., Kleiman E.M., Huang X., Musacchio K.M., Jaroszewski A.C., Chang B.P., Nock M.K. (2017). Risk Factors for Suicidal Thoughts and Behaviors: A Meta-Analysis of 50 Years of Research. Psychol. Bull..

[11] Klonsky E.D., May A.M. (2014). Differentiating Suicide Attempters from Suicide Ideators: A Critical Frontier for Suicidology Research. Suicide Life Threat. Behav..

[12] Gooding P., Pratt D., Pratt D. (2016). Psychological Models of Suicidal Ideation and Behaviour. The Prevention of Suicide in Prison: Cognitive Behavioural Approaches.

[13] Gooding P.A., Harris K., Haddock G. (2022). Psychological Resilience to Suicidal Experiences in People with Non-Affective Psychosis: A Position Paper. Int. J. Environ. Res. Public Health.

[14] Owen R., Jones S.H., Dempsey R.C., Gooding P.A. (2022). Directly or Indirectly? The Role of Social Support in the Psychological Pathways Underlying Suicidal Ideation in People with Bipolar Disorder. Int. J. Environ. Res. Public Health.

[15] O’Connor R.C., Nock M.K. (2014). The Psychology of Suicidal Behaviour. Lancet Psychiatry.

[16] Glenn C.R., Nock M.K. (2014). Improving the Short-Term Prediction of Suicidal Behavior. Am. J. Prev. Med..

[17] Macleod J., Davey Smith G. (2003). Psychosocial Factors and Public Health: A Suitable Case for Treatment?. J. Epidemiol. Community Health.

[18] Zhuang S., Tan D.W., Reddrop S., Dean L., Maybery M., Magiati I. (2023). Psychosocial Factors Associated with Camouflaging in Autistic People and Its Relationship with Mental Health and Well-Being: A Mixed Methods Systematic Review. Clin. Psychol. Rev..

[19] Thomas K., Nilsson E., Festin K., Henriksson P., Lowén M., Löf M., Kristenson M. (2020). Associations of Psychosocial Factors with Multiple Health Behaviors: A Population-Based Study of Middle-Aged Men and Women. Int. J. Environ. Res. Public Health.

[20] Stansfeld S., Rasul F., Steptoe A. (2007). Psychosocial Factors, Depression and Illness. Depression and Physical Illness.

[21] Dempsey R.C., Fedorowicz S.E., Wood A.M. (2023). The Role of Perceived Social Norms in Non-Suicidal Self-Injury and Suicidality: A Systematic Scoping Review. PLoS ONE.

[22] Fedorowicz S.E., Dempsey R.C., Ellis N., Phillips E., Gidlow C. (2023). How Is Suicide Risk Assessed in Healthcare Settings in the UK? A Systematic Scoping Review. PLoS ONE.

[23] Khalil H., Peters M.D., Tricco A.C., Pollock D., Alexander L., McInerney P., Godfrey C.M., Munn Z. (2021). Conducting High Quality Scoping Reviews-Challenges and Solutions. J. Clin. Epidemiol..

[24] Peters M.D.J., Godfrey C.M., Khalil H., McInerney P., Parker D., Soares C.B. (2015). Guidance for Conducting Systematic Scoping Reviews. Int. J. Evid. Based Healthc..

[25] O’Connor R.C., Kirtley O.J. (2018). The Integrated Motivational-Volitional Model of Suicidal Behaviour. Philos. Trans. R. Soc. B Biol. Sci..

[26] Johnson J., Gooding P., Tarrier N. (2008). Suicide Risk in Schizophrenia: Explanatory Models and Clinical Implications, The Schematic Appraisal Model of Suicide (SAMS). Psychol. Psychother. Theory Res. Pract..

[27] Van Orden K.A., Witte T.K., Cukrowicz K.C., Braithwaite S.R., Selby E.A., Joiner T.E. (2010). The Interpersonal Theory of Suicide. Psychol. Rev..

[28] Joiner T.E. (2005). Why People Die by Suicide.

[29] Munn Z., Peters M.D.J., Stern C., Tufanaru C., McArthur A., Aromataris E. (2018). Systematic Review or Scoping Review? Guidance for Authors When Choosing between a Systematic or Scoping Review Approach. BMC Med. Res. Methodol..

[30] Peters M.D.J., Marnie C., Tricco A.C., Pollock D., Munn Z., Alexander L., McInerney P., Godfrey C.M., Khalil H. (2020). Updated Methodological Guidance for the Conduct of Scoping Reviews. JBI Evid. Synth..

[31] Tricco A.C., Lillie E., Zarin W., O’Brien K.K., Colquhoun H., Levac D., Moher D., Peters M.D.J., Horsley T., Weeks L. (2018). PRISMA Extension for Scoping Reviews (PRISMA-ScR): Checklist and Explanation. Ann. Intern. Med..

[32] Ouzzani M., Hammady H., Fedorowicz Z., Elmagarmid A. (2016). Rayyan—A Web and Mobile App for Systematic Reviews. Syst. Rev..

[33] Covidence Systematic Review Software, Veritas Health Innovation, Melbourne, Australia. www.covidence.org.

[34] Page M.J., McKenzie J.E., Bossuyt P.M., Boutron I., Hoffmann T.C., Mulrow C.D., Shamseer L., Tetzlaff J.M., Akl E.A., Brennan S.E. (2021). The PRISMA 2020 Statement: An Updated Guideline for Reporting Systematic Reviews. BMJ.

[35] O’Rourke N., Heisel M.J., Canham S.L., Sixsmith A. (2017). Predictors of Suicide Ideation among Older Adults with Bipolar Disorder. PLoS ONE.

[36] O’Rourke N., Heisel M.J., Canham S.L., Sixsmith A., Yaghoubi-Shahir H., King D.B. (2018). Psychometric Validation of the Geriatric Suicide Ideation Scale (GSIS) among Older Adults with Bipolar Disorder. Aging Ment. Health.

[37] Owen R., Dempsey R., Jones S., Gooding P. (2018). Defeat and Entrapment in Bipolar Disorder: Exploring the Relationship with Suicidal Ideation from a Psychological Theoretical Perspective. Suicide Life Threat. Behav..

[38] Owen R., Gooding P., Dempsey R., Jones S. (2015). A Qualitative Investigation into the Relationships between Social Factors and Suicidal Thoughts and Acts Experienced by People with a Bipolar Disorder Diagnosis. J. Affect. Disord..

[39] Depp C.A., Moore R.C., Dev S.I., Mausbach B.T., Eyler L.T., Granholm E.L. (2016). The Temporal Course and Clinical Correlates of Subjective Impulsivity in Bipolar Disorder as Revealed through Ecological Momentary Assessment. J. Affect. Disord..

[40] Andreu Pascual M., Levenson J.C., Merranko J., Gill M.K., Hower H., Yen S., Strober M., Goldstein T.R., Goldstein B.I., Ryan N.D. (2020). The Effect of Traumatic Events on the Longitudinal Course and Outcomes of Youth with Bipolar Disorder. J. Affect. Disord..

[41] Sewall C.J.R., Girard J.M., Merranko J., Hafeman D., Goldstein B.I., Strober M., Hower H., Weinstock L.M., Yen S., Ryan N.D. (2021). A Bayesian Multilevel Analysis of the Longitudinal Associations between Relationship Quality and Suicidal Ideation and Attempts among Youth with Bipolar Disorder. J. Child. Psychol. Psychiatry.

[42] McGrady A., Lynch D., Rapport D. (2017). Psychosocial Factors and Comorbidity Associated with Suicide Attempts: Findings in Patients with Bipolar Disorder. Psychopathology.

[43] McIntyre R.S., Soczynska J.K., Mancini D., Lam C., Woldeyohannes H.O., Moon S., Konarski J.Z., Kennedy S.H. (2008). The Relationship between Childhood Abuse and Suicidality in Adult Bipolar Disorder. Violence Vict..

[44] Stange J.P., Hamilton J.L., Burke T.A., Kleiman E.M., O’Garro-Moore J.K., Seligman N.D., Abramson L.Y., Alloy L.B. (2015). Negative Cognitive Styles Synergistically Predict Suicidal Ideation in Bipolar Spectrum Disorders: A 3-Year Prospective Study. Psychiatry Res..

[45] Villa J., Pinkham A.E., Kaufmann C.N., Granholm E., Harvey P.D., Depp C.A. (2018). Interpersonal Beliefs Related to Suicide and Facial Emotion Processing in Psychotic Disorders. J. Psychiatr. Res..

[46] Williams J.M.G. (1997). Cry of Pain.

[47] Kapoor S., Freitag S., Bradshaw J., Valencia G.T., Lamis D.A. (2023). The Collective Impact of Childhood Abuse, Psychache, and Interpersonal Needs on Suicidal Ideation among Individuals with Bipolar Disorder: A Discriminant Analysis. Child. Abus. Negl..

[48] Shneidman E.S. (1985). Definition of Suicide.

[49] Lamis D.A., Kapoor S., Evans A.P.B. (2019). Childhood Sexual Abuse and Suicidal Ideation Among Bipolar Patients: Existential But Not Religious Well-Being as a Protective Factor. Suicide Life Threat. Behav..

[50] O’Connor R.C. (2011). The Integrated Motivational-Volitional Model of Suicidal Behaviour. Crisis.

[51] Patton J.H., Stanford M.S., Barratt E.S. (1995). Factor Structure of the Barratt Impulsivity Scale. J. Clin. Psychol..

[52] Aas M., Etain B., Bellivier F., Henry C., Lagerberg T., Ringen A., Agartz I., Gard S., Kahn J.P., Leboyer M. (2014). Additive Effects of Childhood Abuse and Cannabis Abuse on Clinical Expressions of Bipolar Disorders. Psychol. Med..

[53] Aas M., Henry C., Bellivier F., Lajnef M., Gard S., Kahn J.P., Lagerberg T.V., Aminoff S.R., Bjella T., Leboyer M. (2017). Affective Lability Mediates the Association between Childhood Trauma and Suicide Attempts, Mixed Episodes and Co-Morbid Anxiety Disorders in Bipolar Disorders. Psychol. Med..

[54] Aas M., Bellivier F., Bettella F., Henry C., Gard S., Kahn J.P., Lagerberg T.V., Aminoff S.R., Melle I., Leboyer M. (2020). Childhood Maltreatment and Polygenic Risk in Bipolar Disorders. Bipolar Disord..

[55] Adigüzel V., Özdemir N., Şahin Ş.K. (2019). Childhood Traumas in Euthymic Bipolar Disorder Patients in Eastern Turkey and Its Relations with Suicide Risk and Aggression. Nord. J. Psychiatry.

[56] Cazala F., Bauer I.E., Meyer T.D., Spiker D.E., Kazimi I.F., Zeni C.P., Zunta-Soares G.B., Soares J.C. (2019). Correlates of Childhood Trauma in Children and Adolescents with Bipolar Disorder Spectrum: A Preliminary Study. J. Affect. Disord..

[57] Ducasse D., Jaussent I., Guillaume S., Azorin J.M., Bellivier F., Belzeaux R., Bougerol T., Etain B., Gard S., Henry C. (2015). Increased Risk of Suicide Attempt in Bipolar Patients with Severe Tobacco Dependence. J. Affect. Disord..

[58] Etain B., Aas M., Andreassen O.A., Lorentzen S., Dieset I., Gard S., Kahn J.P., Bellivier F., Leboyer M., Melle I. (2013). Childhood Trauma Is Associated with Severe Clinical Characteristics of Bipolar Disorders. J. Clin. Psychiatry.

[59] de Azambuja Farias C., de Azevedo Cardoso T., Mondin T.C., de Mattos Souza L.D., da Silva R.A., Kapczinski F., da Silva Magalhães P.V., Jansen K. (2019). Clinical Outcomes and Childhood Trauma in Bipolar Disorder: A Community Sample of Young Adults. Psychiatry Res..

[60] Garno J.L., Goldberg J.F., Ramirez P.M., Ritzler B.A. (2005). Impact of Childhood Abuse on the Clinical Course of Bipolar Disorder. Br. J. Psychiatry.

[61] Gilbert A.M., Garno J.L., Braga R.J., Shaya Y., Goldberg T.E., Malhotra A.K., Burdick K.E. (2011). Clinical and Cognitive Correlates of Suicide Attempts in Bipolar Disorder: Is Suicide Predictable?. J. Clin. Psychiatry.

[62] Kamali M., Saunders E.F.H., Assari S., Ryan K.A., Marshall D.F., McInnis M.G. (2019). Mood, Dimensional Personality, and Suicidality in a Longitudinal Sample of Patients with Bipolar Disorder and Controls. Suicide Life Threat. Behav..

[63] Grillault Laroche D., Godin O., Dansou Y., Belzeaux R., Aouizerate B., Burté T., Courtet P., Dubertret C., Haffen E., Llorca P.M. (2022). Influence of Childhood Maltreatment on Prevalence, Onset, and Persistence of Psychiatric Comorbidities and Suicide Attempts in Bipolar Disorders. Eur. Psychiatry.

[64] Lima I.M.M., Malloy-Diniz L.F., de Miranda D.M., da Silva A.G., Neves F.S., Johnson S.L. (2017). Integrative Understanding of Familial Impulsivity, Early Adversity and Suicide Risk. Front. Psychol..

[65] Nobile B., Dubois J., Aouizerate B., Aubin V., Loftus J., Bellivier F., Belzeaux R., Dubertret C., Gard S., Haffen E. (2021). Characterization of Depressed Bipolar Patients with Current Suicidal Ideation. Aust. N. Z. J. Psychiatry.

[66] Pavlova B., Perroud N., Cordera P., Uher R., Alda M., Dayer A., Aubry J.M. (2018). Anxiety Disorders and Childhood Maltreatment as Predictors of Outcome in Bipolar Disorder. J. Affect. Disord..

[67] Segura A.G., Mitjans M., Jiménez E., Fatjó-Vilas M., Ruiz V., Saiz P.A., García-Portilla M.P., González-Blanco L., Bobes J., Vieta E. (2019). Association of Childhood Trauma and Genetic Variability of CRH-BP and FKBP5 Genes with Suicidal Behavior in Bipolar Patients. J. Affect. Disord..

[68] Xie P., Wu K., Zheng Y., Guo Y., Yang Y., He J., Ding Y., Peng H. (2018). Prevalence of Childhood Trauma and Correlations between Childhood Trauma, Suicidal Ideation, and Social Support in Patients with Depression, Bipolar Disorder, and Schizophrenia in Southern China. J. Affect. Disord..

[69] Yilmaz O., Ateş M.A., Semiz B., Tütüncü R., Bez Y., Algül A., Balibey H., Başoğlu C., Ebrinç S., Çetin M. (2016). Childhood Traumas in Patients with Bipolar Disorder: Association with Alexithymia and Dissociative Experiences. Anadolu Psikiyatr. Derg..

[70] Jaworska-Andryszewska P., Rybakowski J.K. (2018). Childhood Adversity and Clinical Features of Bipolar Mood Disorder. Arch. Psychiatry Psychother..

[71] Mert D.G., Kelleci M., Mizrak A., Semiz M., Demir M.O. (2015). Factors Associated with Suicide Attempts in Patients with Bipolar Disorder Type I. Psychiatr. Danub..

[72] Cakir S., Tasdelen Durak R., Ozyildirim I., Ince E., Sar V. (2016). Childhood Trauma and Treatment Outcome in Bipolar Disorder. J. Trauma. Dissoc..

[73] Freitag S., Kapoor S., Lamis D.A. (2022). Childhood Maltreatment, Impulsive Aggression, and Suicidality Among Patients Diagnosed with Bipolar Disorder. Psychol. Trauma.

[74] Janiri D., Sani G., Danese E., Simonetti A., Ambrosi E., Angeletti G., Erbuto D., Caltagirone C., Girardi P., Spalletta G. (2015). Childhood Traumatic Experiences of Patients with Bipolar Disorder Type i and Type II. J. Affect. Disord..

[75] Larsson S., Aas M., Klungsøyr O., Agartz I., Mork E., Steen N.E., Barrett E.A., Lagerberg T.V., Røssberg J.I., Melle I. (2013). Patterns of Childhood Adverse Events Are Associated with Clinical Characteristics of Bipolar Disorder. BMC Psychiatry.

[76] Manoli A., Wright L.C., Shakoor S., Fisher H.L., Hosang G.M. (2023). The Association between Childhood Bullying Victimisation and Childhood Maltreatment with the Clinical Expression of Bipolar Disorder. J. Psychiatr. Res..

[77] Guillen-Burgos H., Moreno-Lopez S., Acevedo-Vergara K., Pérez-Florez M., Pachón-Garcia C., Gálvez-Flórez J.F. (2023). Risk of Childhood Trauma Exposure and Severity of Bipolar Disorder in Colombia. Int. J. Bipolar Disord..

[78] Zhang Y., Hu Z., Hu M., Lu Z., Yu H., Yuan X. (2022). Effects of Childhood Trauma on Nonsuicidal Self-Injury in Adolescent Patients with Bipolar II Depression. Brain Behav..

[79] Colic L., Clark A., Sankar A., Rathi D.J., Goldman D.A., Kim J.A., Villa L.M., Edmiston E.K., Lippard E.T.C., Pittman B. (2022). Gender-Related Association among Childhood Maltreatment, Brain Structure and Clinical Features in Bipolar Disorder. Eur. Neuropsychopharmacol..

[80] Caruso D., Palagini L., Miniati M., Massa L., Marazziti D., Geoffroy P.A., Etain B. (2021). Early Life Stress and Chronobiological Rhythms Desynchronization: Possible Impact on Mood Symptoms and Suicidal Ideation in Bipolar Disorder. J. Nerv. Ment. Dis..

[81] Ivković M., Pantović-Stefanović M., Dunjić-Kostić B., Jurišić V., Lačković M., Totić-Poznanović S., Jovanović A.A., Damjanović A. (2016). Neutrophil-to-Lymphocyte Ratio Predicting Suicide Risk in Euthymic Patients with Bipolar Disorder: Moderatory Effect of Family History. Compr. Psychiatry.

[82] Palagini L., Miniati M., Marazziti D., Sharma V., Riemann D. (2021). Association among Early Life Stress, Mood Features, Hopelessness and Suicidal Risk in Bipolar Disorder: The Potential Contribution of Insomnia Symptoms. J. Psychiatr. Res..

[83] Palagini L., Miniati M., Marazziti D., Franceschini C., Zerbinati L., Grassi L., Sharma V., Riemann D. (2022). Insomnia Symptoms Are Associated with Impaired Resilience in Bipolar Disorder: Potential Links with Early Life Stressors May Affect Mood Features and Suicidal Risk. J. Affect. Disord..

[84] Marwaha S., Briley P.M., Perry A., Rankin P., Diflorio A., Craddock N., Jones I., Broome M., Gordon-Smith K., Jones L. (2020). Explaining Why Childhood Abuse Is a Risk Factor for Poorer Clinical Course in Bipolar Disorder: A Path Analysis of 923 People with Bipolar i Disorder. Psychol. Med..

[85] Erten E., Funda Uney A., Saatçioǧlu Ö., Özdemir A., Fistikçi N., Çakmak D. (2014). Effects of Childhood Trauma and Clinical Features on Determining Quality of Life in Patients with Bipolar i Disorder. J. Affect. Disord..

[86] Johnson S.L., Carver C.S., Tharp J.A. (2017). Suicidality in Bipolar Disorder: The Role of Emotion-Triggered Impulsivity. Suicide Life Threat. Behav..

[87] Fijtman A., Clausen A., Kauer-Sant’Anna M., Morey R. (2023). Trauma History in Veterans with Bipolar Disorder and Its Impact on Suicidality. J. Psychiatr. Res..

[88] Park Y.M., Shekhtman T., Kelsoe J.R. (2020). Effect of the Type and Number of Adverse Childhood Experiences and the Timing of Adverse Experiences on Clinical Outcomes in Individuals with Bipolar Disorder. Brain Sci..

[89] Masi G., Lupetti I., D’acunto G., Milone A., Fabiani D., Madonia U., Berloffa S., Lenzi F., Mucci M. (2021). A Comparison between Severe Suicidality and Nonsuicidal Self-Injury Behaviors in Bipolar Adolescents Referred to a Psychiatric Emergency Unit. Brain Sci..

[90] Leverich G.S., McElroy S.L., Suppes T., Keck P.E., Denicoff K.D., Nolen W.A., Altshuler L.L., Rush A.J., Kupka R., Frye M.A. (2002). Early Physical and Sexual Abuse Associated with an Adverse Course of Bipolar Illness. Biol. Psychiatry.

[91] Leverich G.S., Altshuler L.L., Frye M.A., Suppes T., Keck P.E., McElroy S.L., Denicoff K.D., Obrocea G., Nolen W.A., Kupka R. (2003). Factors Associated with Suicide Attempts in 648 Patients with Bipolar Disorder in the Stanley Foundation Bipolar Network. J. Clin. Psychiatry.

[92] Du Rocher Schudlich T., Youngstrom E.A., Martinez M., KogosYoungstrom J., Scovil K., Ross J., Feeny N.C., Findling R.L. (2015). Physical and Sexual Abuse and Early-Onset Bipolar Disorder in Youths Receiving Outpatient Services: Frequent, but Not Specific. J. Abnorm. Child. Psychol..

[93] Kamali M., Reilly-Harrington N.A., Chang W.C., McInnis M., McElroy S.L., Ketter T.A., Shelton R.C., Deckersbach T., Tohen M., Kocsis J.H. (2019). Bipolar Depression and Suicidal Ideation: Moderators and Mediators of a Complex Relationship. J. Affect. Disord..

[94] Sewall C.J.R., Goldstein T.R., Salk R.H., Merranko J., Gill M.K., Strober M., Keller M.B., Hafeman D., Ryan N.D., Yen S. (2020). Interpersonal Relationships and Suicidal Ideation in Youth with Bipolar Disorder. Arch. Suicide Res..

[95] Perich T., Mitchell P.B., Loo C., Hadzi-Pavlovic D., Roberts G., Green M., Frankland A., Lau P., Corry J. (2014). Cognitive Styles and Clinical Correlates of Childhood Abuse in Bipolar Disorder. Bipolar Disord..

[96] Goldstein T.R., Birmaher B., Axelson D., Ryan N.D., Strober M.A., Gill M.K., Valeri S., Chiappetta L., Leonard H., Hunt J. (2005). History of Suicide Attempts in Pediatric Bipolar Disorder: Factors Associated with Increased Risk. Bipolar Disord..

[97] Kauer-Sant’Anna M., Tramontina J., Andreazza A.C., Cereser K., da Costa S., Santin A., Yatham L.N., Kapczinski F. (2007). Traumatic Life Events in Bipolar Disorder: Impact on BDNF Levels and Psychopathology. Bipolar Disord..

[98] Carballo J.J., Harkavy-Friedman J., Burke A.K., Sher L., Baca-Garcia E., Sullivan G.M., Grunebaum M.F., Parsey R.V., Mann J.J., Oquendo M.A. (2008). Family History of Suicidal Behavior and Early Traumatic Experiences: Additive Effect on Suicidality and Course of Bipolar Illness?. J. Affect. Disord..

[99] Khoubaeva D., Dimick M., Timmins V.H., Fiksenbaum L.M., Mitchell R.H.B., Schaffer A., Sinyor M., Goldstein B.I. (2023). Clinical Correlates of Suicidality and Self-Injurious Behaviour among Canadian Adolescents with Bipolar Disorder. Eur. Child. Adolesc. Psychiatry.

[100] Khosravani V., Berk M., Sharifi Bastan F., Samimi Ardestani S.M., Wrobel A. (2021). The Effects of Childhood Emotional Maltreatment and Alexithymia on Depressive and Manic Symptoms and Suicidal Ideation in Females with Bipolar Disorder: Emotion Dysregulation as a Mediator. Int. J. Psychiatry Clin. Pract..

[101] Grunebaum M.F., Ramsay S.R., Galfalvy H.C., Ellis S.P., Burke A.K., Sher L., Printz D.J., Kahn D.A., Mann J.J., Oquendo M.A. (2006). Correlates of Suicide Attempt History in Bipolar Disorder: A Stress-Diatheasis Perspective. Bipolar Disord..

[102] Oquendo M.A., Waternaux C., Brodsky B., Parsons B., Haas G.L., Malone K.M., Mann J.J. (2000). Suicidal Behavior in Bipolar Mood Disorder: Clinical Characteristics of Attempters and Nonattempters. J. Affect. Disord..

[103] av Kák Kollsker S., Coello K., Stanislaus S., Melbye S., Lie Kjærstad H., Stefanie Ormstrup Sletved K., Vedel Kessing L., Vinberg M. (2022). Association between Lifetime and Recent Stressful Life Events and the Early Course and Psychopathology in Patients with Newly Diagnosed Bipolar Disorder, First-Degree Unaffected Relatives and Healthy Controls: Cross-Sectional Results from a Prospective St. Bipolar Disord..

[104] Azorin J.M., Kaladjian A., Adida M., Hantouche E., Hameg A., Lancrenon S., Akiskal H.S. (2009). Risk Factors Associated with Lifetime Suicide Attempts in Bipolar I Patients: Findings from a French National Cohort. Compr. Psychiatry.

[105] Özer S., Uluşahin A., Batur S., Kabakçi E., Saka M.C. (2002). Outcome Measures of Interepisode Bipolar Patients in Turkish Sample. Soc. Psychiatry Psychiatr. Epidemiol..

[106] Vai B., Serretti A., Poletti S., Mascia M., Lorenzi C., Colombo C., Benedetti F. (2020). Cortico-Limbic Functional Connectivity Mediates the Effect of Early Life Stress on Suicidality in Bipolar Depressed 5-HTTLPR*s Carriers. J. Affect. Disord..

[107] Benedetti F., Riccaboni R., Poletti S., Radaelli D., Locatelli C., Lorenzi C., Pirovano A., Smeraldi E., Colombo C. (2014). The Serotonin Transporter Genotype Modulates the Relationship between Early Stress and Adult Suicidality in Bipolar Disorder. Bipolar Disord..

[108] Benedetti F., Riccaboni R., Dallaspezia S., Locatelli C., Smeraldi E., Colombo C. (2015). Effects of CLOCK Gene Variants and Early Stress on Hopelessness and Suicide in Bipolar Depression. Chronobiol. Int..

[109] Subramanian K., Menon V., Sarkar S., Chandrasekaran V., Selvakumar N. (2020). Study of Risk Factors Associated with Suicide Attempt in Patients with Bipolar Disorder Type I. J. Neurosci. Rural. Pract..

[110] Antypa N., Antonioli M., Serretti A. (2013). Clinical, Psychological and Environmental Predictors of Prospective Suicide Events in Patients with Bipolar Disorder. J. Psychiatr. Res..

[111] Mazaheri M., Gharraee B., Shabani A., Lotfi M. (2019). Studying the Predictive Factors of Suicide Attempts in Patients with Type 1 Bipolar Disorder. Psychiatry Res..

[112] Goldstein T.R., Birmaher B., Axelson D., Goldstein B.I., Gill M.K., Esposito-Smythers C., Ryan N.D., Strober M.A., Hunt J., Keller M. (2009). Family Environment and Suicidal Ideation Among Bipolar Youth. Arch. Suicide Res..

[113] Barratt E.S. (1959). Anxiety and Impulsiveness Related to Psychomotor Efficiency. Percept. Mot. Ski..

[114] Bezerra-Filho S., Galvão-de-Almeida A., Studart P., Martins D.F., Caribé A.C., Schwingel P.A., Miranda-Scippa Â. (2017). Suicide Attempts in Bipolar I Patients: Impact of Comorbid Personality Disorders. Rev. Bras. Psiquiatr..

[115] Caribé A.C., Studart P., Bezerra-Filho S., Brietzke E., Nunes Noto M., Vianna-Sulzbach M., Kapczinski F., Silva Neves F., Correa H., Miranda-Scippa Â. (2015). Is Religiosity a Protective Factor against Suicidal Behavior in Bipolar I Outpatients?. J. Affect. Disord..

[116] Etain B., Mathieu F., Liquet S., Raust A., Cochet B., Richard J.R., Gard S., Zanouy L., Kahn J.P., Cohen R.F. (2013). Clinical Features Associated with Trait-Impulsiveness in Euthymic Bipolar Disorder Patients. J. Affect. Disord..

[117] Feki I., Moalla M., Baati I., Trigui D., Sellami R., Masmoudi J. (2016). Impulsivity in Bipolar Disorders in a Tunisian Sample. Asian J. Psychiatr..

[118] Henna E., Hatch J.P., Nicoletti M., Swann A.C., Zunta-Soares G., Soares J.C. (2013). Is Impulsivity a Common Trait in Bipolar and Unipolar Disorders?. Bipolar Disord..

[119] Izci F., Zincir S., Zincir S., Bilici R., Yalcin M., Terzi A., Goncu T., Akdur O., Gica S., Semiz Ü.B. (2015). Temperament-Character Features, Levels of Impulsiveness and Functionality in Bipolar Disorder Patients with and without Suicide Attempts: A Controlled Study. J. Mood Disord..

[120] Jiménez E., Arias B., Mitjans M., Goikolea J.M., Ruíz V., Brat M., Sáiz P.A., García-Portilla M.P., Burón P., Bobes J. (2016). Clinical Features, Impulsivity, Temperament and Functioning and Their Role in Suicidality in Patients with Bipolar Disorder. Acta Psychiatr. Scand..

[121] Kahn J.P., Cohen R.F., Etain B., Aubin V., Bellivier F., Belzeaux R., Bougerol T., Courtet P., Dubertret C., Gard S. (2019). Reconsideration of the Factorial Structure of the Barratt Impulsiveness Scale (BIS-11): Assessment of Impulsivity in a Large Population of Euthymic Bipolar Patients. J. Affect. Disord..

[122] Lage R.R., de Assis da Silva R., Tancini M.B., Nardi A.E., Mograbi D.C., Cheniaux E. (2022). Suicidal Ideation in Bipolar Disorder: The Relation with Clinical and Sociodemographic Variables. Psychiatr. Q..

[123] Masi G., Scullin S., Narzisi A., Muratori P., Paciello M., Fabiani D., Lenzi F., Mucci M., D’acunto G. (2020). Suicidal Ideation and Suicidal Attempts in Referred Adolescents with High Functioning Autism Spectrum Disorder and Comorbid Bipolar Disorder: A Pilot Study. Brain Sci..

[124] Matsuo K., Nielsen N., Nicoletti M.A., Hatch J.P., Monkul E.S., Watanabe Y., Zunta-Soares G.B., Nery F.G., Soares J.C. (2010). Anterior Genu Corpus Callosum and Impulsivity in Suicidal Patients with Bipolar Disorder. Neurosci. Lett..

[125] Michaelis B.H., Goldberg J.F., Singer T.M., Garno J.L., Ernst C.L., Davis G.P. (2003). Characteristics of First Suicide Attempts in Single versus Multiple Suicide Attempters with Bipolar Disorder. Compr. Psychiatry.

[126] Michaelis B.H., Goldberg J.F., Davis G.P., Singer T.M., Garno J.L., Wenze S.J. (2004). Dimensions of Impulsivity and Aggression Associated with Suicide Attempts Among Bipolar Patients: A Preliminary Study. Suicide Life Threat. Behav..

[127] Ostacher M.J., LeBeau R.T., Perlis R.H., Nierenberg A.A., Lund H.G., Moshier S.J., Sachs G.S., Simon N.M. (2009). Cigarette Smoking Is Associated with Suicidality in Bipolar Disorder. Bipolar Disord..

[128] Parmentier C., Etain B., Yon L., Misson H., Mathieu F., Lajnef M., Cochet B., Raust A., Kahn J.P., Wajsbrot-Elgrabli O. (2012). Clinical and Dimensional Characteristics of Euthymic Bipolar Patients with or without Suicidal Behavior. Eur. Psychiatry.

[129] Perroud N., Baud P., Mouthon D., Courtet P., Malafosse A. (2011). Impulsivity, Aggression and Suicidal Behavior in Unipolar and Bipolar Disorders. J. Affect. Disord..

[130] Sublette M.E., Carballo J.J., Moreno C., Galfalvy H.C., Brent D.A., Birmaher B., Mann J.J., Oquendo M.A. (2009). Substance Use Disorders and Suicide Attempts in Bipolar Subtypes. J. Psychiatr. Res..

[131] Swann A.C., Lijffijt M., Lane S.D., Steinberg J.L., Gerard Moeller F.G. (2009). Increased Trait-like Impulsivity and Course of Illness in Bipolar Disorder. Bipolar Disord..

[132] Swann A.C., Steinberg J.L., Lijffijt M., Moeller F.G. (2008). Impulsivity: Differential Relationship to Depression and Mania in Bipolar Disorder. J. Affect. Disord..

[133] Umamaheswari V., Avasthi A., Grover S. (2014). Risk Factors for Suicidal Ideations in Patients with Bipolar Disorder. Bipolar Disord..

[134] Zakowicz P., Skibińska M., Wasicka-Przewoźna K., Skulimowski B., Waśniewski F., Chorzepa A., Różański M., Twarowska-Hauser J., Pawlak J. (2021). Impulsivity as a Risk Factor for Suicide in Bipolar Disorder. Front. Psychiatry.

[135] Ekinci O., Albayrak Y., Ekinci A.E., Caykoylu A. (2011). Relationship of Trait Impulsivity with Clinical Presentation in Euthymic Bipolar Disorder Patients. Psychiatry Res..

[136] Swann A.C., Dougherty D.M., Pazzaglia P.J., Pham M., Steinberg J.L., Moeller F.G. (2005). Increased Impulsivity Associated with Severity of Suicide Attempt History in Patients with Bipolar Disorder. Am. J. Psychiatry.

[137] Reich R., Gilbert A., Clari R., Burdick K.E., Szeszko P.R. (2019). A Preliminary Investigation of Impulsivity, Aggression and White Matter in Patients with Bipolar Disorder and a Suicide Attempt History. J. Affect. Disord..

[138] Kulacaoglu F., Izci F. (2022). The Effect of Emotional Dysregulation and Impulsivity on Suicidality in Patients with Bipolar Disorder. Psychiatr. Danub..

[139] Şenormancı G., Güçlü O., Özben İ., Karakaya F.N., Şenormancı Ö. (2020). Resilience and Insight in Euthymic Patients with Bipolar Disorder. J. Affect. Disord..

[140] Edge M.D., Johnson S.L., Ng T., Carver C.S. (2013). Iowa Gambling Task Performance in Euthymic Bipolar i Disorder: A Meta-Analysis and Empirical Study. J. Affect. Disord..

[141] Stanford M.S., Houston R.J., Mathias C.W., Villemarette-Pittman N.R., Helfritz L.E., Conklin S.M. (2003). Characterizing Aggressive Behavior. Assessment.

[142] Russo J.M., Naclerio M., Kaplan C., Cho E., Lee E., Salisbury A., Au J.S., Tirpak J.W., Dickstein D.P. (2023). Sensation Seeking in Children and Adults with Pediatric-Onset Bipolar Disorder. Child. Psychiatry Hum. Dev..

[143] Swann A.C., Lijffijt M., Lane S.D., Steinberg J.L., Moeller F.G. (2009). Severity of Bipolar Disorder Is Associated with Impairment of Response Inhibition. J. Affect. Disord..

[144] Malloy-Diniz L.F., Neves F.S., De Moraes P.H.P., De Marco L.A., Romano-Silva M.A., Krebs M.O., Corrêa H. (2011). The 5-HTTLPR Polymorphism, Impulsivity and Suicide Behavior in Euthymic Bipolar Patients. J. Affect. Disord..

[145] de Almeida V.F., Bezerra-Filho S., Studart-Bottó P., Léda-Rego G., Silva I.T.F., Kapczinski F., Miranda-Scippa Â. (2021). History of Suicide Attempts in Patients with Bipolar Disorder Type I: Socio-Demographic and Clinical Factors, Quality of Life and Functioning. Nord. J. Psychiatry.

[146] Karantonis J.A., Rossell S.L., Berk M., Rheenen T.E. (2021). Van the Mental Health and Lifestyle Impacts of COVID-19 on Bipolar Disorder. J. Affect. Disord..

[147] Palagini L., Miniati M., Caruso D., Cappelli A., Massa L., Pardini F., Petrucci A., Romeo F., Salarpi G., Etain B. (2021). Predictors of Suicidal Ideation and Preparatory Behaviors in Individuals with Bipolar Disorder: The Contribution of Chronobiological Dysrhythmicity and Its Association with Hopelessness. J. Clin. Psychiatry.

[148] Kazan Kizilkurt O., Giynas F.E., Yazici Gulec M., Gulec H. (2019). Bipolar Disorder and Perceived Social Support: Relation with Clinical Course, and the Role of Suicidal Behaviour. Psychiatry Clin. Psychopharmacol..

[149] Studart P., Galvão-de Almeida A., Bezerra-Filho S., Caribé A., Reis Afonso N., Daltro C., Miranda-Scippa A. (2016). Is History of Suicidal Behavior Related to Social Support and Quality of Life in Outpatients with Bipolar I Disorder?. Psychiatry Res..

[150] Valtonen H.M., Suominen K., Mantere O., Leppämäki S., Arvilommi P., Isometsä E.T. (2006). Prospective Study of Risk Factors for Attempted Suicide among Patients with Bipolar Disorder. Bipolar Disord..

[151] Valtonen H., Suominen K., Mantere O., Leppämäki S., Arvilommi P., Isometsä E.T. (2005). Suicidal Ideation and Attempts in Bipolar I and II Disorders. J. Clin. Psychiatry.

[152] Acosta J.R., Librenza-Garcia D., Watts D., Francisco A.P., Zórtea F., Raffa B., Kohmann A., Mugnol F.E., Motta G.L., Tramontina S. (2020). Bullying and Psychotic Symptoms in Youth with Bipolar Disorder. J. Affect. Disord..

[153] Şen O., Yildizhan E. (2020). Relationship of Intolerance of Uncertainty and Attachment Styles with the Clinical Features of Bipolar Disorder in Remission. Turk. Psikiyatr. Derg..

[154] Tsai S.Y., Lee J.C., Chen C.C. (1999). Characteristics and Psychosocial Problems of Patients with Bipolar Disorder at High Risk for Suicide Attempt. J. Affect. Disord..

[155] Dervic K., Carballo J.J., Baca-Garcia E., Galfalvy H.C., Mann J.J., Brent D.A., Oquendo M.A. (2008). Moral or Religious Objections to Suicide May Protect Against Suicidal Behavior in Bipolar Disorder. J. Clin. Psychiatry.

[156] Daskalopoulou E.G., Dikeos D.G., Papadimitriou G.N., Souery D., Blairy S., Massat I., Mendlewicz J., Stefanis C.N. (2002). Self-Esteem, Social Adjustment and Suicidality in Affective Disorders. Eur. Psychiatry.

[157] Li Y.C., Bai W., Cai H., Wu Y., Zhang L., Ding Y.H., Yang J.J., Du X., Zeng Z.T., Lu C.M. (2022). Suicidality in Clinically Stable Bipolar Disorder and Schizophrenia Patients during the COVID-19 Pandemic. Transl. Psychiatry.

[158] Beck A.T., Weissman A., Lester D., Trexler L. (1974). The Measurement of Pessimism: The Hopelessness Scale. J. Consult. Clin. Psychol..

[159] Acosta F.J., Vega D., Torralba L., Navarro S., Ramallo-Fariña Y., Fiuza D., Hernández J.L., Siris S.G. (2012). Hopelessness and Suicidal Risk in Bipolar Disorder. A Study in Clinically Nonsyndromal Patients. Compr. Psychiatry.

[160] Allen M.H., Chessick C.A., Miklowitz D.J., Goldberg J.F., Wisniewski S.R., Miyahara S., Calabrese J.R., Marangell L., Bauer M.S., Thomas M.R. (2005). Contributors to Suicidal Ideation Among Bipolar Patients with and Without a History of Suicide Attempts. Suicide Life Threat. Behav..

[161] Ekşioğlu S., Güleç H., Semiz Ü.B., Şimşek G. (2015). The Relationship of Suicide Attempts with Affective Temperament and Relevant Clinical Features in Patients with Mood Disorders. Turk. Psikiyatr. Derg..

[162] Marangell L.B., Bauer M.S., Dennehy E.B., Wisniewski S.R., Allen M.H., Miklowitz D.J., Oquendo M.A., Frank E., Perlis R.H., Martinez J.M. (2006). Prospective Predictors of Suicide and Suicide Attempts in 1556 Patients with Bipolar Disorders Followed for up to 2 Years. Bipolar Disord..

[163] Pompili M., Rihmer Z., Akiskal H., Amore M., Gonda X., Innamorati M., Lester D., Perugi G., Serafini G., Telesforo L. (2012). Temperaments Mediate Suicide Risk and Psychopathology among Patients with Bipolar Disorders. Compr. Psychiatry.

[164] Valtonen H., Suominen K., Haukka J., Mantere O., Leppamaki S., Arvilommi P., Isometsa E. (2008). Differences in Incidence of Suicide Attempts during Phases of Bipolar I and II Disorders. Bipolar Disord..

[165] Valtonen H.M., Suominen K., Mantere O., Leppämäki S., Arvilommi P., Isometsä E. (2007). Suicidal Behaviour during Different Phases of Bipolar Disorder. J. Affect. Disord..

[166] Rucklidge J.J. (2006). Psychosocial Functioning of Adolescents with and without Paediatric Bipolar Disorder. J. Affect. Disord..

[167] Weinstein S.M., Van Meter A., Katz A.C., Peters A.T., West A.E. (2015). Cognitive and Family Correlates of Current Suicidal Ideation in Children with Bipolar Disorder. J. Affect. Disord..

[168] Kazdin A.E., Rodgers A., Colbus D. (1986). The Hopelessness Scale for Children: Psychometric Characteristics and Concurrent Validity. J. Consult. Clin. Psychol..

[169] Bauer M.S., Wisniewski S.R., Marangell L.B., Chessick C.A., Allen M.H., Dennehy E.B., Miklowitz D.J., Thase M.E., Sachs G.S. (2006). Are Antidepressants Associated with New-Onset Suicidality in Bipolar Disorder? A Prospective Study of Participants in the Systematic Treatment Enhancement Program for Bipolar Disorder (STEP-BD). J. Clin. Psychiatry.

[170] Ekinci O., Ekinci A. (2013). An Investigation of the Three Factor Model of Personality and Its Relationships with Clinical Characteristics in Major Mood Disorders. Noropsikiyatri Ars..

[171] Kudinova A.Y., MacPherson H.A., Musella K., Schettini E., Gilbert A.C., Jenkins G.A., Clark L.A., Dickstein D.P. (2021). Maladaptive Personality Traits and the Course of Suicidal Ideation in Young Adults with Bipolar Disorder: Cross-Sectional and Prospective Approaches. Suicide Life Threat. Behav..

[172] Clark L., Simms L., Wu K., Casillas A. (2014). Schedule for Nonadaptive and Adaptive Personality-2nd Edition (SNAP-2): Manual for Administration, Scoring, and Interpretation.

[173] Engström C., Brändström S., Sigvardsson S., Cloninger C.R., Nylander P.O. (2004). Bipolar Disorder. III: Harm Avoidance a Risk Factor for Suicide Attempts. Bipolar Disord..

[174] Erić A.P., Erić I., Ćurković M., Dodig-Ćurković K., Kralik K., Kovač V., Filaković P. (2017). The Temperament and Character Traits in Patients with Major Depressive Disorder and Bipolar Affective Disorder with and without Suicide Attempt. Psychiatr. Danub..

[175] Sarisoy G., Kaçar Ö.F., Pazvantoǧlu O., Öztürk A., Korkmaz I.Z., Kocamanoǧlu B., Böke Ö., Sahin A.R. (2012). Temperament and Character Traits in Patients with Bipolar Disorder and Associations with Attempted Suicide. Compr. Psychiatry.

[176] Fico G., Caivano V., Zinno F., Carfagno M., Steardo L., Sampogna G., Luciano M., Fiorillo A. (2019). Affective Temperaments and Clinical Course of Bipolar Disorder: An Exploratory Study of Differences among Patients with and without a History of Violent Suicide Attempts. Medicina.

[177] Henry C., Lacoste J., Bellivier F., Verdoux H., Bourgeois M.L., Leboyer M. (1999). Temperament in Bipolar Illness: Impact on Prognosis. J. Affect. Disord..

[178] Fico G., Janiri D., Pinna M., Sagué-Vilavella M., Gimenez Palomo A., Oliva V., De Prisco M., Cortez P.G., Anmella G., Gonda X. (2023). Affective Temperaments Mediate Aggressive Dimensions in Bipolar Disorders: A Cluster Analysis from a Large, Cross-Sectional, International Study. J. Affect. Disord..

[179] Fernandez E., Day A., Boyle G.J. (2015). Measures of Anger and Hostility in Adults.

[180] The WHOQOL Group (1998). Development of the World Health Organization WHOQOL-BREF Quality of Life Assessment. Psychol. Med..

[181] De Abreu L.N., Nery F.G., Harkavy-Friedman J.M., De Almeida K.M., Gomes B.C., Oquendo M.A., Lafer B. (2012). Suicide Attempts Are Associated with Worse Quality of Life in Patients with Bipolar Disorder Type I. Compr. Psychiatry.

[182] Türk A., Uğurlu N.B. (2023). Internalized Stigma and the Quality of Life and Self-Esteem of Individuals with Bipolar Disorder. J. Psychiatr. Nurs..

[183] Endicott J., Nee J., Harrison W., Blumenthal R. (1993). Quality of Life Enjoyment and Satisfaction Questionnaire: A New Measure. Psychopharmacol. Bull..

[184] Ravens-Sieberer U., Bullinger M. (2000). KINDL-R: Questionnaire for Measuring Health-Related Quality of Life in Children and Adolescents–Revised.

[185] Algorta G.P., Youngstrom E.A., Frazier T.W., Freeman A.J., Findling R.L., Youngstrom J.K. (2011). Suicidality in Pediatric Bipolar Disorder: Predictor or Outcome of Family Processes and Mixed Mood Presentation?. Bipolar Disord..

[186] Gruhn M.A., West A., Hamlat E., Weinstein S. (2021). Assessing Change in Suicidal Ideation Intensity for Youth in Treatment for Pediatric Bipolar Disorder. Clin. Child. Psychol. Psychiatry.

[187] Eissa M.F., Elghoniemy S., Hamed D., Omar A.N., Morsy M. (2012). The Quality of Life in Patients with Bipolar Disorder Who Have Achieved Remission in an Egyptian Sample. Middle East. Curr. Psychiatry.

[188] Hasse-Sousa M., Martins D.S., Petry-Perin C., Arrial-Cordeiro R.T., Rabelo-da-Ponte F.D., Rosa A.R., Bücker J., Gama C.S., Czepielewski L.S. (2020). Performance in Inhibitory Control during Euthymia Is Not Related to Past Suicide Attempts in Individuals with Bipolar Disorder. Eur. J. Psychiatry.

[189] Luo X., Zhu Y., Lu D., Zong K., Lin X. (2020). Subjective Cognitive Dysfunction in Patients with Bipolar Disorder: The Prevalence, Related Factors and Effects on Predicting Psychosocial Functioning and Suicidal Ideation. Psychiatry Res..

[190] Lima F.M., Cardoso T.A., Serafim S.D., Martins D.S., Solé B., Martínez-Arán A., Vieta E., Rosa A.R. (2018). Validity and Reliability of the Cognitive Complaints in Bipolar Disorder Rating Assessment (COBRA) in Brazilian Bipolar Patients. Trends Psychiatry Psychother..

[191] Malloy-Diniz L.F., Neves F.S., Abrantes S.S.C., Fuentes D., Corrêa H. (2009). Suicide Behavior and Neuropsychological Assessment of Type I Bipolar Patients. J. Affect. Disord..

[192] Martino D.J., Strejilevich S.A., Torralva T., Manes F. (2011). Decision Making in Euthymic Bipolar i and Bipolar II Disorders. Psychol. Med..

[193] MacPherson H.A., Kudinova A.Y., Schettini E., Jenkins G.A., Gilbert A.C., Thomas S.A., Kim K.L., Radoeva P.D., Fenerci R.L.B., Yen S. (2022). Relationship between Cognitive Flexibility and Subsequent Course of Mood Symptoms and Suicidal Ideation in Young Adults with Childhood-Onset Bipolar Disorder. Eur. Child. Adolesc. Psychiatry.

[194] Martínez-Arán A., Vieta E., Reinares M., Colom F., Torrent C., Sánchez-Moreno J., Benabarre A., Goikolea J.M., Comes M., Salamero M. (2004). Cognitive Function Across Manic or Hypomanic, Depressed, and Euthymic States in Bipolar Disorder. Am. J. Psychiatry.

[195] Yen C.F., Cheng C.P., Ko C.H., Yen J.Y., Huang C.F., Chen C.S. (2008). Suicidality and Its Association with Insight and Neurocognition in Taiwanese Patients with Bipolar I Disorder in Remission. J. Nerv. Ment. Dis..

[196] Broadbent D.E., Cooper P.F., FitzGerald P., Parkes K.R. (1982). The Cognitive Failures Questionnaire (CFQ) and Its Correlates. Br. J. Clin. Psychol..

[197] Aksoy Poyraz C., Özdemir A., Şen C.Ç., Usta Sağlam N.G., Enginkaya S., Tomruk N. (2021). The Impact of Coping Strategies on Suicide Attempts and Suicidal Ideation in Bipolar Disorder. J. Nerv. Ment. Dis..

[198] Simon N.M., Pollack M.H., Ostacher M.J., Zalta A.K., Chow C.W., Fischmann D., Demopulos C.M., Nierenberg A.A., Otto M.W. (2007). Understanding the Link between Anxiety Symptoms and Suicidal Ideation and Behaviors in Outpatients with Bipolar Disorder. J. Affect. Disord..

[199] Stroppa A., Moreira-Almeida A. (2013). Religiosity, Mood Symptoms, and Quality of Life in Bipolar Disorder. Bipolar Disord..

[200] Gratz K.L., Roemer L. (2004). Multidimensional Assessment of Emotion Regulation and Dysregulation: Development, Factor Structure, and Initial Validation of the Difficulties in Emotion Regulation Scale. J. Psychopathol. Behav. Assess..

[201] Palagini L., Miniati M., Marazziti D., Massa L., Grassi L., Geoffroy P.A. (2022). Circadian Rhythm Alterations May Be Related to Impaired Resilience, Emotional Dysregulation and to the Severity of Mood Features in Bipolar I and II Disorders. Clin. Neuropsychiatry.

[202] Goldstein T.R., Merranko J., Rode N., Sylvester R., Hotkowski N., Fersch-Podrat R., Hafeman D.M., Diler R., Sakolsky D., Franzen P. (2023). Dialectical Behavior Therapy for Adolescents with Bipolar Disorder. JAMA Psychiatry.

[203] Palagini L., Cipollone G., Moretto U., Masci I., Tripodi B., Caruso D., Perugi G. (2019). Chronobiological Dis-Rhythmicity Is Related to Emotion Dysregulation and Suicidality in Depressive Bipolar II Disorder with Mixed Features. Psychiatry Res..

[204] Dell’osso L., Cremone I.M., Amatori G., Cappelli A., Cuomo A., Barlati S., Massimetti G., Vita A., Fagiolini A., Carmassi C. (2021). Investigating the Relationship between Autistic Traits, Ruminative Thinking, and Suicidality in a Clinical Sample of Subjects with Bipolar Disorder and Borderline Personality Disorder. Brain Sci..

[205] Ellis A.J., Portnoff L.C., Axelson D.A., Kowatch R.A., Walshaw P., Miklowitz D.J. (2014). Parental Expressed Emotion and Suicidal Ideation in Adolescents with Bipolar Disorder. Psychiatry Res..

[206] Berutti M., Dias R.S., Pereira V.A., Lafer B., Nery F.G. (2016). Association between History of Suicide Attempts and Family Functioning in Bipolar Disorder. J. Affect. Disord..

[207] Khosravani V., Mohammadzadeh A., Sharifi Bastan F., Amirinezhad A., Amini M. (2019). Early Maladaptive Schemas and Suicidal Risk in Inpatients with Bipolar Disorder. Psychiatry Res..

[208] Nilsson K.K. (2016). Early Maladaptive Schemas in Bipolar Disorder Patients with and without Suicide Attempts. J. Nerv. Ment. Dis..

[209] Cesur E., Şahmelikoğlu Onur Ö., Erten E. (2019). Differences in Metacognitive Beliefs among Patients with Bipolar Disorder with or without Previous Suicide Attempts. Nord. J. Psychiatry.

[210] de Assis da Silva R., Mograbi D.C., Bifano J., Santana C.M.T., Cheniaux E. (2017). Correlation Between Insight Level and Suicidal Behavior/Ideation in Bipolar Depression. Psychiatr. Q..

[211] de Assis da Silva R., Mograbi D.C., Camelo E.V.M., Santana C.M.T., Landeira-Fernandez J., Cheniaux E. (2017). Clinical Correlates of Loss of Insight in Bipolar Depression. Trends Psychiatry Psychother..

[212] Linehan M.M., Goodstein J.L., Nielsen S.L., Chiles J.A. (1983). Reasons for Staying Alive When You Are Thinking of Killing Yourself: The Reasons for Living Inventory. J. Consult. Clin. Psychol..

[213] Choi J.W., Cha B., Jang J., Park C.S., Kim B.J., Lee C.S., Lee S.J. (2015). Resilience and Impulsivity in Euthymic Patients with Bipolar Disorder. J. Affect. Disord..

[214] Fernández-Rocha M.L., García-Izquierdo M., Ríos-Rísquez M.I. (2021). Psychological Resilience and Suicide Attempt in Patients With Bipolar Disorder: An Exploratory Study. J. Am. Psychiatr. Nurses Assoc..

[215] Karaytuğ M.O., Tamam L., Demirkol M.E., Namlı Z., Gürbüz M., Yeşiloğlu C., Eriş Davut Ö. (2022). The Mediating Role of Time Perspective in the Relationship between Chronotype and Suicide in Bipolar Disorder. Behav. Sci..

[216] Wood A.M., Tarrier N. (2010). Positive Clinical Psychology: A New Vision and Strategy for Integrated Research and Practice. Clin. Psychol. Rev..

[217] Ten Have M., De Graaf R., Van Dorsselaer S., Verdurmen J., Van’t Land H., Vollebergh W., Beekman A. (2009). Incidence and Course of Suicidal Ideation and Suicide Attempts in the General Population. Can. J. Psychiatry.

[218] Mars B., Heron J., Klonsky E.D., Moran P., O’Connor R.C., Tilling K., Wilkinson P., Gunnell D. (2019). Predictors of Future Suicide Attempt among Adolescents with Suicidal Thoughts or Non-Suicidal Self-Harm: A Population-Based Birth Cohort Study. Lancet Psychiatry.

[219] Arksey H., O’Malley L. (2005). Scoping Studies: Towards a Methodological Framework. Int. J. Soc. Res. Methodol..

